# Childhood maltreatment disrupts HPA-axis activity under basal and stress conditions in a dose–response relationship in children and adolescents

**DOI:** 10.1017/S003329172100249X

**Published:** 2023-02

**Authors:** Laia Marques-Feixa, Helena Palma-Gudiel, Soledad Romero, Jorge Moya-Higueras, Marta Rapado-Castro, Águeda Castro-Quintas, Iñaki Zorrilla, María José Muñoz, Maite Ramírez, María Mayoral, Ariadna Mas, María José Lobato, Hilario Blasco-Fontecilla, Lourdes Fañanás, EPI-Young Stress GROUP, María Martín, Eulalia Anglada, Pilar Santamarina, Patricia Rubio, Iría Méndez, Dolores Moreno, María Ramos, Jorge Vidal, Juanjo Carballo, Elena Font, Lydia Gayubo, Laura Colino, María Rodrigo-Yanguas, Maddi Laborde, Jaume March-Llanes

**Affiliations:** 1 Department of Evolutionary Biology, Ecology and Environmental Sciences, Faculty of Biology, University of Barcelona, Biomedicine Institute of the University of Barcelona (IBUB), Barcelona, Spain; 2 Network Centre for Biomedical Research in Mental Health (CIBER of Mental Health, CIBERSAM), Spain; 3 Department of Child and Adolescent Psychiatry and Psychology, Institute of Neuroscience, Hospital Clínic de Barcelona, 2017SGR88, Barcelona, Spain; 4 Institut d'Investigacions Biomediques August Pi i Sunyer (IDIBAPS), Barcelona, Spain; 5 Department of Psychology, Faculty of Education, Psychology and Social Work, University of Lleida, Spain; 6 Department of Child and Adolescent Psychiatry, Institute of Psychiatry and Mental Health, Hospital General Universitario Gregorio Marañón, School of Medicine, Universidad Complutense, IiSGM, Madrid, Spain; 7 Department of Psychiatry, Melbourne Neuropsychiatry Centre, The University of Melbourne & Melbourne Health, Victoria, Australia; 8 Department of Psychiatry, Hospital Santiago Apostol, Vitoria-Gasteiz, Spain; 9 Hospital Benito Menni, Adolescent Crisis Unit, Sant Boi de Llobregat, Spain; 10 Galdakao Mental Health Services, Child and Adolescent Mental Health, Galdakao, Spain; 11 Department of Psychiatry, Puerta de Hierro University Hospital-Majadahonda, Autonoma University, ITA Mental Health, Madrid, Spain; 12 Day Hospital for Adolescents, Fundació Orienta, Gavà, Spain

**Keywords:** Anxiety perception, child abuse, childhood maltreatment (CM), cortisol, dose–response, hypothalamic–pituitary–adrenal (HPA)-axis, Trier Social Stress Test for children (TSST-C), youth psychopathology

## Abstract

**Background:**

This study investigates the impact of childhood maltreatment (CM) on hypothalamic–pituitary–adrenal (HPA)-axis functioning and on anxiety perception. Moreover, the influence of CM severity and frequency was also explored.

**Methods:**

In total, 187 participants aged 7–17 were assessed for CM history using validated questionnaires and *ad hoc* interviews to be classified according to the criteria of the Tool for Assessing the Severity of Situations in which Children are Vulnerable (TASSCV). Psychopathology was ascertained using the K-SADS-PL5. To assess HPA-axis functioning, salivary cortisol samples were collected throughout a normal day and during an acute psychosocial stressor, the Trier Social Stress Test for children (TSST-C). Subjective anxiety was evaluated using STAI/-C.

**Results:**

Youth with a CM history had higher overall diurnal cortisol levels (*p* = 0.001), blunted cortisol response to acute psychosocial stress (*p* = 0.002) and greater perceived anxiety (*p* = 0.003), than those without CM. Specifically, participants exposed to moderate/severe or often/frequent CM showed the greater diurnal cortisol output (*p*_severity_ = 0.002; *p*_frequency_ = 0.003), and blunted cortisol response during the TSST-C (*p*_severity_ = 0.006; *p*_frequency_ = 0.008). Meanwhile, youth with low CM severity/frequency exhibited a similar cortisol response to those without CM. However, perceived anxiety was higher in those exposed to CM (*p* < 0.001), regardless of its severity/frequency.

**Conclusions:**

Disturbances in HPA-axis functioning are already evident early after CM exposure, while psychological and physiological responses to an acute stressor are dissociated in youth exposed to CM. The dose–response relationship described in this paper highlights the need to comprehensively evaluate CM so that vulnerable children can be identified and assigned to proper interventions.

## Introduction

Experiences of childhood maltreatment (CM) are one of the main contributors to mental illness (Brown, Harris, & Craig, 2019; Hughes et al., 2017). However, CM is non-specifically associated with psychiatric disorders, i.e. several types of CM can increase vulnerability for a specific disorder in different patients (Vachon, Krueger, Rogosch, & Cicchetti, 2015). CM has been associated with early onset of psychiatric illness, increased symptom severity and comorbidity, and poor clinical outcomes characterized by requiring higher medication dosages, increased suicidal behavior, and more and longer hospitalizations (Lippard & Nemeroff, 2020). Furthermore, factors such as time of exposure, chronicity, and severity of childhood abuse or neglect play a role in clinical outcomes. Studies indicate a dose–response relationship between multiplicity of exposure, severity or frequency, and risk of mental disorders (Anda et al., 2006).

CM is associated with dysregulation of stress-mediating systems, thereby increasing the risk of mental and physical health problems. Specifically, disruptions in hypothalamic–pituitary–adrenal (HPA)-axis regulation have been studied as a potential mediator of this association (Koss & Gunnar, 2018; Kudielka & Wüst, 2010). The HPA-axis is one of the main stress response systems; cortisol, its final effector, released in direct response to acute stressors, triggers a wide range of actions by regulating gene transcription and epigenetic modifications in several brain areas (Provençal, Arloth, Cattaneo, Anacker, & Cattane, 2019). Furthermore, HPA-axis maintains a diurnal rhythm, with the highest cortisol levels in the morning which decrease progressively during the day until reaching the lowest at midnight. Since the HPA-axis continues to mature during early stages of life, environmental factors such as early-life stress may induce long-lasting changes in its functioning, resulting in the emergence of different disorders (Tarullo & Gunnar, 2006). However, findings regarding alterations in the patterns of cortisol associated with early-life stress have been inconsistent (Fogelman & Canli, 2018).

A recent meta-analysis focusing on CM and diurnal HPA-axis activity in children and adults reported no overall effect on diurnal cortisol slope (Bernard, Frost, Bennett, & Lindhiem, 2017). However, a moderate association was found between CM and blunted awakening cortisol concentrations when considering only sufferers of CM who were referred from child welfare system agencies. In contrast, another recent meta-analysis showed that CM affects HPA-axis reactivity during stressful situations as evidenced by a flattened cortisol pattern during an acute psychosocial stress task in children and adults who faced early-life adversities (Bunea, Szentágotai-t, & Miu, 2017). Interestingly, the effects were more pronounced in studies focused on adults and CM. These findings suggest a pattern of blunted cortisol response during the peak and recovery phases of acute stress, and overall hypocortisolism in individuals exposed to CM. However, some studies report hypercortisolism in subjects exposed to early-life stress, childhood trauma, or insensitive interactions with caregivers (Hunter, Minnis, & Wilson, 2011). Besides, it has been suggested that distinct patterns of cortisol responses may be partially explained by CM severity and frequency (Ouellet-Morin et al., 2019), pubertal stage (King et al., 2017), or sex (Trickett, Gordis, Peckins, & Susman, 2014).

Notably, HPA-axis dysregulation, both hyperactivity and hypoactivity, has been associated with different psychiatric disorders and other disease outcomes (Turner et al., 2020). Although infancy is a sensitive period for HPA-axis regulation, this system remains plastic and it can be recalibrated during specific ontogenic periods, if the environmental conditions improve. In fact, recent studies support puberty as a key recalibration period to trigger shifts in HPA-axis functioning in postinstitutionalized children (DePasquale, Donzella, & Gunnar, 2019).

Thus, the main aim of the current research was to establish the proximal effects of CM on HPA-axis regulation and anxiety perception in children and adolescents, under basal conditions and in response to a psychosocial stressor, as compared with youth without CM. In addition, the differential impact of the severity and frequency of the CM experiences was also analyzed to better dissect the relationship between CM and HPA-axis dysfunction. Finally, anxiety perception was assessed throughout the experimental stress paradigm to verify that all participants underwent a subjective experience of stress (regardless of their CM history); thus, the potential differences in stress perception with regard to CM can be disentangled from actual differences in HPA-axis functioning. Complementarily, anxiety trait was also assessed in relationship with basal diurnal cortisol output. Specifically, we hypothesized that exposure to CM would be associated with blunted HPA-axis functioning and higher anxiety perception. Moreover, more severe and frequent exposure to CM would be associated with greater dysregulation of the HPA-axis following a dose–response relationship.

## Methods

The EPI-Young-Stress project is a multi-center study which aims to evaluate HPA-axis functioning, associated epigenetic signatures, and immunological biomarkers involved in the association between CM and youth mental disorders. The research was conducted at the University of Barcelona and six child and adolescent psychiatry departments in Spain: Hospital Benito Menni, Hospital Clínic Barcelona, Hospital Gregorio Marañón, Hospital Puerta de Hierro, Hospital Santiago Apóstol, and Day Hospital Orienta Gavà.

The study was approved by the Ethical Review Board of each participating hospital and university. Families were explicitly informed about the voluntary nature of the study, their rights, and the procedures, risks, and potential benefits involved. Written consent was required from all parents or legal guardians; the children provided written assent after the nature of the procedure had been fully explained.

### Participants

A total of 187 children and adolescents aged 7–17 years participated in this study. Children without psychopathology were recruited from advertisements, primary healthcare centers, schools, and other community facilities. Children with current psychopathology were recruited from the above-mentioned hospitals (inpatient clinics, partial hospitalization programs, and outpatient clinics) (see Table 1). Recruitment lasted from April 2016 to March 2020. Exclusion criteria for all participants included diagnosis of autism spectrum disorder, eating disorder with body mass index (BMI)<18.5, intellectual disability (IQ < 70), current drug dependence, non-fluency in Spanish, extreme premature birth (<1500 g at birth), head injury with loss of consciousness, and severe neurological or other pathological conditions likely to affect HPA-axis functioning (such as cancer or autoimmune diseases).

**Table 1.** Sociodemographic and anthropometric data of participants with and without a history of CM

Variable			Total sample (*n* = 187)	Youth without CM (*n* = 93, 50%)	Youth with CM (*n* = 94, 50%)	*t*/χ^2^	*p*	*d*/*κ*
Age (M, s.d.)^a^			13.62 (2.59)	13.20 (2.69)	14.03 (2.44)	−2.204	0.029*	0.323
Sex^b^	Female (*n*, %)		108 (58%)	48 (52%)	60(64%)	2.860	0.091	0.122
	Male (*n*, %)		79 (42%)	45 (48%)	34 (36%)			
Pubertal stage^b^	Child (Tanner stage 1–3) (*n*, %)		94 (50%)	53 (57%)	41 (44%)	3.344	0.067	0.134
	Adolescent (Tanner stage 4–5) (*n*, %)		93 (50%)	40 (43%)	53 (56%)			
Ethnicity^b^	European (*n*, %)		154 (82%)	87 (93%)	67 (71%)	15.956	<0.001***	0.222
	Others^c^ (*n*, %)		33 (18%)	6 (7%)	27 (29%)			
Socioeconomic status (SES) (M, s.d.)^a,d^			40.34 (17.93)	47.49 (14.77)	33.12 (18.03)	5.893	<0.001***	0.872
CGAS (M, s.d.)^a^			72.07 (21.66)	84.26 (14.37)	59.88 (20.89)	9.270	<0.001***	1.359
Current psychiatric diagnosis status^b^	Subjects without current psychiatric diagnosis (*n*, %)		71 (38%)	56 (60%)	15 (16%)	38.879	<0.001***	−0.442
	Subjects with current psychiatric diagnosis (*n*, %):		116 (62%)	37 (40%)	79 (84%)			
	Primary psychiatric diagnosis dimensions^b,e^	ADHD	30 (26%)	18 (49%)	12 (15%)	32.235	<0.001***	0.119
		Affective disorders	29 (25%)	6 (16%)	23 (29%)			
		Trauma and stress-related disorders	19 (16%)	0 (0%)	19 (24%)			
		Anxiety disorders	15 (13%)	9 (24%)	6 (8%)			
		Behavioral disorders	13 (11%)	1 (3%)	12 (15%)			
		Psychotic disorders	7 (6%)	3 (8%)	4 (5%)			
		Eating disorders	3 (3%)	0 (0%)	3 (4%)			
	Clinical care units of subjects with current psychiatric diagnosis^b,e^	Outpatient	69 (60%)	31 (83%)	38 (48%)	13.458	0.001**	−0.262
		Inpatient	35 (30%)	5 (14%)	30 (38%)			
		Partial program	12 (10%)	1 (3%)	11 (14%)			
	Psychopharmacological treatment of subjects with current psychiatric diagnosis^b,e^	No (*n*, %)	28 (24%)	9 (24%)	19 (24%)	0.001	0.974	<0.001
		Yes (*n*, %)	88 (76%)	28 (76%)	60 (76%)			
Oral contraceptive use^b,f^	No (*n*, %)		102 (94%)	47 (98%)	55 (92%)	1.985	0.159	0.056
	Yes (*n*, %)		6 (6%)	1 (2%)	5 (8%)			
Corticosteroid medication^b^	No (*n*, %)		184 (98%)	90 (97%)	94 (100%)	3.082	0.079	−0.032
	Yes (*n*, %)		3 (2%)	3 (3%)	0 (0%)			
Last year global family environmental (GFES) (M, s.d.)^a,g^			78.24 (15.03)	84.53 (9.65)	71.94 (16.76)	6.104	<0.001***	0.920
Illegal drug use^b^	Never		164 (88%)	90 (97%)	74 (79%)	15.242	0.002**	0.124
	Less than once a month		10 (5%)	1 (1%)	9 (10%)			
	Once a month or more		7 (4%)	2 (2%)	5 (5%)			
	Daily use		6 (3%)	0 (0%)	6 (6%)			
BMI (M, s.d.)^a,h^			21.45 (5.17)	19.66 (3.75)	23.23 (5.77)	−4.799	<0.001***	0.733
WHR (M, s.d.)^a,h^			0.84 (0.09)	0.84 (0.09)	0.84 (0.09)	0.059	0.953	−0.011

ADHD, attention-deficit/hyperactivity disorder; BMI, body mass index; CGAS, Children's Global Assessment Scale, rating from 1 to 100 with higher ratings indicating better functioning in a wide range of activities; CM, childhood maltreatment (CM group refers to subjects with a confirmed or suspected history of CM); GFES, The Global Family Environment Scale, ranging from 1 to 90, with higher scores indicating a better family environment; SES, socioeconomic status, raw scores range from 8 to 66, with higher scores reflecting higher SES; WHR, waist-to-hip ratio.

a Student's *t* test.

b χ^2^ test.

c Other ethnicities included Latin American (66%), Maghrebin (16%), sub-Saharan (9%), and others (9%).

^d^This analysis was conducted with 183 subjects.

^e^This analysis was only conducted with the 116 subjects with a current psychiatric diagnosis.

^f^This analysis was only conducted with the 108 female subjects.

^g^This analysis was conducted with 176 subjects.

^h^This analysis was conducted within 171 subjects.

*p* values: *p < 0.05, **p < 0.01, and ***p < 0.001. *d* = Cohen's effect size.

### Procedures

#### Sociodemographic and clinical measures

The interview package included basic demographic information including socioeconomic status (SES) based on the Hollingshead Four-Factor Index of SES (Hollingshead, 1975). Pubertal development was assessed using the Tanner staging questionnaire (Morris & Udry, 1980) and participants were classified as either children (Tanner stages 1–3) or adolescents (Tanner stages 4–5). The Global Family Environment Scale (GFES) was used to measure the quality of the family environment (Rey et al., 1997). Additionally, ethnicity, BMI and waist-to-hip ratio were recorded.

Both participants and their parents directly recounted the youth's medical history. Psychopathology was assessed using the Spanish version of the Schedule for Affective Disorders and Schizophrenia for School-Age Children Present and Lifetime Version DSM-5 (K-SADS-PL-5) (de la Peña et al., 2018). Information was completed whenever possible using medical records. Final diagnoses were established by consensus, and based on DSM-5 criteria (APA: American Psychiatric Association, 2013), primary psychiatric diagnoses were later classified into dimensions to better characterize the sample (see Table 1). The global level of functioning was measured by the Children's Global Assessment Scale (CGAS) (Shaffer et al., 1983). The use of psychiatric medication was dichotomized as absence/presence, since there were no differences in cortisol levels according to the different drugs (data available upon request). Current illegal drug use was classified into four frequency groups: never, less than once a month, once a month or more, and daily use (Forti et al., 2019).

#### Childhood maltreatment assessment

All participants and their parents/legal guardians were interviewed separately, face to face, by one trained psychologist or psychiatrist. They were assessed by means of an exhaustive interview focused on the identification of signs of child vulnerability, adverse experiences, and family interactions, based on the criteria of the instrument ‘Tool for assessing the severity of situations in which children are vulnerable’ (TASSCV), which has been validated by professionals working in child and adolescent care units (see online Supplementary material) (CARM, 2012). Additionally, adolescents older than 12 were assessed for history of CM via the short version of the Childhood Trauma Questionnaire (CTQ-SF) (Bernstein et al., 2003) and the Childhood Experience of Care and Abuse Questionnaire (CECA-Q2) (Kaess et al., 2011). Children under 12 years answered an adapted hetero-administered *ad hoc* questionnaire (see online Supplementary material). Afterwards, the clinicians completed a table summarizing the different forms of CM effected by caregivers or other adults (not by peers), being TASSCV the main measure of CM used in the primary analyses, while the other measures (CTQ-SF, CECA-Q2, *ad hoc* questionnaire, and reports from social services or teachers) were used as an additional source of information for the clinicians. The exhaustive participants' evaluation during the recruitment process allowed for clinicians to enrich their praxis. In addition, after the interviews of this study, a referral system of urgent appointment was implemented for those subjects who requested it, activating the usual protocols that guarantee the children's protection rights. Following the TASSCV criteria, each CM type was coded as either: (i) absent, (ii) suspected (if significant signs of neglect or abuse emerged during the evaluation), or (iii) confirmed (with clear evidence from social services or family). Severity and frequency of different types of CM were rated on a four-point Likert scale according to TASSCV criteria. CM severity was coded according to the characteristics of the experience suffered as low (1), moderate (2), severe (3), or very severe (4); while frequency was coded as whether CM had occurred once (1), sometimes (2), often (3), or frequently (4). Five types of CM were considered in the following analysis: physical neglect, emotional neglect, physical abuse, emotional abuse, and sexual abuse.

#### HPA-axis functioning

**Fig. 1.**
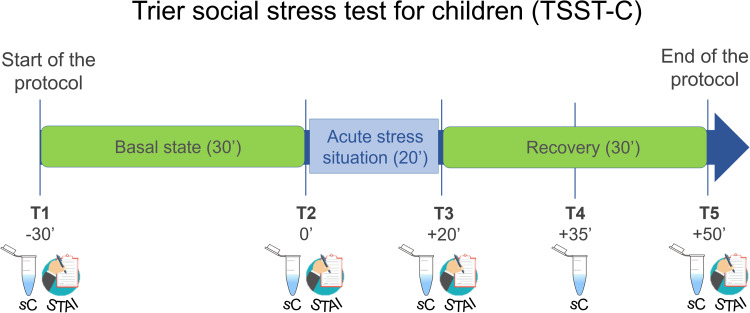
Summary of the Trier Social Stress Test for children (TSST-C) protocol. sC, salivary cortisol sample; STAI, State/Trait Anxiety Inventory – State.

Four saliva samples were collected during a normal day with the aim to assess HPA-axis diurnal functioning (basal condition), specifically, on waking up (B1), 30 min after waking (B2), before lunch (B3), and before bedtime (B4). On a different day, in order to explore HPA-axis reactivity during acute psychosocial stress, the Trier Social Stress Test for children (TSST-C), a validated protocol that reliably induces HPA-axis activation, was applied (Buske-Kirschbaum et al., 1997). Briefly, upon arrival at the lab, the participants waited in a quiet room for 30 min before entering the examination room, where a panel of judges awaited. During the 20 min of the stress situation, the participants had to perform a speaking and an arithmetic task following instructions from the judges while being videotaped. After the stress task, the participants returned to the first room for 30 min (see online Supplementary material for a more detailed description of the procedure). Five saliva samples were collected during this procedure: 30 min before the stressor (T1), immediately before the stressor (T2), immediately after the stressor (T3), 15 min after the stressor (T4), and 30 min after the stressor (T5) (see Fig. 1). All participants were scheduled at 16:00 h to control for diurnal cortisol variability. Previously, further instructions were given to the participants to avoid factors that have been reported to influence cortisol levels (details in the online Supplementary material). Details about collection time of each salivary cortisol sample are available in Table 2.

**Table 2.** Cortisol values and anxiety perception according to the presence of CM, CM severity and CM frequency

		Dichotomous CM (mean, s.d.)		Severity of CM (mean, s.d.)		Frequency of CM (mean, s.d.)		*F* (*p*) dichotomous CM^a^	*F* (*p*) severity of CM^a^	*F* (*p*) frequency of CM ^a^
		Youth without CM (*n* = 93)	Youth with CM (*n* = 94)	Youth with low CM (*n* = 20)	Youth with moderate/severe CM (*n* = 74)	Youth once/sometimes exposed to CM (*n* = 22)	Youth often-frequently exposed to CM (*n* = 72)			
Diurnal salivary cortisol (μm log-transformed)	B1	−0.66 (0.32)	−0.66 (0.25)	−0.63 (0.24)	−0.67 (0.25)	−0.67 (0.20)	−0.66 (0.26)	1.467 (0.225) ^B4*^	1.214 (0.300) ^B4*^	1.085 (0.375) ^B4*^
	B2	−0.51 (0.31)	−0.52 (0.31)	−0.44 (0.23)	−0.54 (0.33)	−0.40 (0.19)	−0.56 (0.34)			
	B3	−1.09 (0.29)	−1.03 (0.34)	−0.93 (0.34)	−1.05 (0.33)	−0.95 (0.34)	−1.05 (0.34)			
	B4	−1.56 (0.59)	−1.38 (0.45)	−1.46 (0.35)	−1.36 (0.47)	−1.39 (0.38)	−1.38 (0.48)			
	AUCg	−927.77 (262.45)	−831.64 (202.25)	−799.16 (221.60)	−839.42 (198.39)	−765.10 (194.54)	−851.22 (201.84)	**12.244** (0.001**) 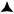	6.349 (0.002**) 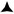	6.068 (0.003**) 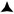
	AUCi	−374.70 (326.25)	−291.13 (246.56)	−297.62 (261.08)	−289.58 (244.88)	−234.17 (229.26)	−307.89 (250.57)	3.040 (0.083)	1.716 (0.184)	1.276 (0.282)
Salivary cortisol during TSST-C (μm log-transformed)	T1	−0.97 (0.31)	−0.97 (0.26)	−0.99 (0.30)	−0.96 (0.25)	−0.97 (0.26)	−0.97 (0.26)	**4.530** (0.002**) ^T3*, T4**, T5*^	**2.773** (0.006**) ^T3*, T4**, T5*^	**2.665** (0.008**) ^T3*, T4**, T5*^
	T2	−1.02 (0.26)	−1.01 (0.27)	−1.01 (0.24)	−1.00 (0.28)	−1.01 (0.23)	−1.00 (0.29)			
	T3	−0.89 (0.31)	−0.98 (0.27)	−0.93 (0.25)	−0.99 (0.29)	−0.95 (0.26)	−0.99 (0.29)			
	T4	−0.89 (0.35)	−1.03 (0.32)	−0.99 (0.33)	−1.04 (0.32)	−1.01 (0.30)	−1.03 (0.32)			
	T5	−0.98 (0.35)	−1.07 (0.31)	−1.09 (0.28)	−1.07 (0.32)	−1.07 (0.26)	−1.07 (0.32)			
	AUCg	−75.22 (21.74)	−78.67 (18.75)	−80.52 (22.87)	−78.26 (17.84)	−80.90 (18.94)	−78.02 (18.77)	0.091 (0.763)	0.057 (0.945)	0.074 (0.929)
	AUCi	0.56 (15.55)	−2.79 (15.02)	24 (17.24)	−3.47 (145.3)	−2.19 (16.51)	−2.97 (14.69)	**4.779** (0.030*)	**3.921** (0.022*)	**3.194** (0.044*)
Anxiety trait: STAI-Trait (PC)		36.98 (28.40)	65.39 (32.74)	60.06 (31.50)	66.69 (33.12)	56.10 (34.14)	68.16 (32.04)	**9.129** (0.003**) 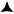	**5.109** (0.007**)	**4.102** (0.019*)
Perceived anxiety during TSST-C: STAI-State (PC)	T1	25.68 (27.90)	45.34 (33.88)	46.59 (33.13)	45.05 (34.25)	32.05 (28.59)	49.27 (34.50)	1.742 (0.160)	1.670 (0.131)	1.240 (0.287)
	T2	25.20 (27.40)	40.40 (33.04)	32.07 (27.83)	42.07 (33.92)	30.05 (29.99)	43.64 (33.50)			
	T3	43.68 (32.53)	66.58 (32.18)	56.76 (36.70)	68.80 (30.90)	61.62 (32.16)	68.04 (32.27)			
	T5	21.12 (27.42)	42.49 (35.08)	29.41 (34.09)	45.50 (34.84)	27.14 (30.21)	47.10 (35.33)			

AUCg, area under the curve with respect to ground (indicating the total cortisol output); AUCi, area under the curve with respect to increase (reflecting cortisol changes over time); CM, childhood maltreatment (CM group refers to the subjects with a confirmed or suspected history of CM based on TASSCV criteria); STAI-State (PC), percentile scores of state anxiety inventory scale (for adolescents 16–17 years old) and state anxiety inventory for children scale (for participants under 15); STAI-Trait (PC), percentile scores of anxiety trait inventory scale (for adolescents 16–17 years old) and anxiety trait inventory for children scale (for participants under 15); TSST-C, Trier Social Stress Test for children.

Diurnal salivary cortisol was measured at: B1, immediately after awakening; B2, 30 min after waking; B3, before lunch; B4, before bedtime. Mean time for saliva sample collection: 08:52 ± 1:27 (6:00–12:00) (B1); 09:24 ± 1:26 (6:30–12:59) (B2); 14:19 ± 0:53 (12:15–16:40) (B3); and 22:37 ± 01:16 [20:00–2:50(+1day)] (B4). Saliva samples for cortisol measurement during TSST-C were collected at: T1, 30 min before stressor; T2, immediately before stressor; T3, immediately after stressor; T4, 15 min after stressor; T5, 30 min after stressor. Mean time for saliva sample collection during the TSST-C procedure: 16:04 ± 0:11 (15:13–17:15) (T1); 16:33 ± 0:12 (15:42–17:45) (T2); 16:53 ± 0:13 (15:59–18:00) (T3); 17:08 ± 0:13 (16:08-18:16) (T4); and 17:23 ± 0:13 (16:30–18:30)(T5).

Dichotomous CM refers to the analysis comparing youth without CM with youth exposed to any type of CM. Severity of CM refers to the analysis comparing youth without CM, youth exposed to low CM, and youth exposed to moderate/severe CM. Frequency of CM refers to the analysis comparing youth without CM, youth exposed to CM once/sometimes, and youth exposed to CM often/frequently.

a Mixed-effects model (for single measurements) and ANOVA (for AUCg and AUCi). The analyses include the following covariates: clinical status, sex, pubertal stage, psychopharmacological treatment, illegal drugs use, oral contraceptive use, corticosteroid medication, ethnicity, SES, and BMI [additionally adjusting by the time of the first cortisol sample collection (B1) for diurnal analysis]. Values in superscript ^(B4, T3, T4, T5)^ indicate the samples with a significant difference in the simple effects test in the context of mixed-effect model.

*p* values: *p < 0.05, **p < 0.01, and ***p < 0.001. 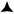
*p* ⩽ 0.006 [as the Bonferroni-corrected level of significance for multiple testing (0.05/9 = 0.006)].

Saliva samples were collected using Salivette^®^ tubes (Sarstedt, Inc., Newton, NC, USA) for diurnal cortisol assessment and with Saliva Bio Oral Swabs (SOS) (Salimetrics, LLC, State College, PA, USA) for TSST-C cortisol reactivity. The subjects were asked to chew a swab for 1 min and then transfer it directly from their mouth to the tube. They were instructed to store their saliva samples for diurnal cortisol assessment in a freezer until they could be delivered to the research center, where samples were stored at −20 °C. The saliva samples collected during the TSST-C were directly stored at the research center. Details of salivary cortisol determination procedures are explained in the online Supplementary material.

#### Anxiety trait and anxiety perception during acute stress

The subscale trait of the State-Trait Anxiety Inventory (STAI) was used to evaluate general proneness to anxious behavior (STAI-Trait for children, for subjects 15 years old and under; STAI-Trait, for adolescents 16–17 years old) (Spielberger, 1973). During the TSST-C, the perceived emotional arousal was assessed via the STAI-State for children scale (for children 15 and under) and the STAI-State subscale (for adolescents 16–17 years old) (Spielberger, 1973). Participants answered the STAI-State questionnaire: 30 min before the stressor (T1), immediately before the stressor (T2), immediately after the stressor (T3), and 30 min after the stressor (T5) (see Fig. 1).

### Statistical analysis

All statistical analyses were performed using SPSS 26 for Windows (IBM, Chicago, Illinois, USA). Descriptive statistics were analyzed by Student's *t* test for continuous variables and a χ^2^ test for categorical variables. Cortisol data were log-transformed to reduce skewness. The presence of any type of suspected or confirmed history of CM was included in downstream analysis as a dichotomic variable. The effects of both (i) CM severity (classified as: absent, low, or moderate/severe) and (ii) the frequency of CM (classified as: never, once/sometimes, or often/frequently) were also tested through independent analyses. Sensitivity analysis was conducted to explore the effects of CM when considering only subjects with a confirmed history of CM (with clear evidence from social service reports or family), aggregating those with suspected history of CM together with those without CM (see online Supplementary material).

To examine the effect of CM in diurnal cortisol slopes and changes in cortisol and anxiety perception across the TSST-C, mixed-effects models with a random effect of intercept and a random slope of time, to account for within-subject correlations, were used. Interaction with time was considered the main effect of interest of the model. Time factor had four categories (time-points) for diurnal cortisol and anxiety perception during TSST-C, and five categories for cortisol during TSST-C. In addition, simple effects tests were performed to evaluate the specific time point interaction between groups. Additionally, the overall cortisol secretion during a normal day and throughout the experimental protocol was summarized applying: (i) the area under the curve with respect to ground (AUCg) to explore the total hormonal output, and (ii) the area under the curve with respect to increase (AUCi) to reflect hormonal changes over time (Pruessner, Kirschbaum, Meinlschmidt, & Hellhammer, 2003). Differences in AUCg, AUCi, and STAI-Trait scores between CM groups were tested by ANOVA. All the analyses were adjusted for the following covariates, as previously described to influence cortisol output during the TSST (Allen, Kennedy, Cryan, Dinan, & Clarke, 2014; De Punder, Heim, & Entringer, 2019; Lê-scherban et al., 2018; Marceau & Abel, 2018): clinical status, sex, pubertal stage, psychopharmacological treatment, illegal drugs use, oral contraceptive use, corticosteroid medication, ethnicity, SES, and BMI. In the diurnal cortisol analyses, the time of first cortisol sample (B1) collection was also included as a covariate. Specifically, in the ANOVA analysis, in order to study the direct effect of clinical status, sex, and pubertal stage on cortisol and anxiety, as well as their potential interactions with CM, these variables were included as inter-subject factors. To correct for the testing of three different CM variables (presence/absence of CM, CM severity, and CM frequency) and three different cortisol summary measures (mixed model, AUCg, and AUCi), in Table 2, a Bonferroni correction was applied by dividing the original *α* level (*p* < 0.05) by 9 (3 × 3), and obtained a new significance level of *p* < 0.006. Spearman's non-parametric correlation was calculated separately in participants without CM and those with a history of CM, to explore the relationship between anxiety perception and salivary cortisol during basal conditions and during the TSST-C.

## Results

### Attrition and descriptive analysis

Nine subjects had no information available on diurnal cortisol levels, so they were not included in the diurnal cortisol analysis. Three participants had no information available on cortisol and anxiety perception during the TSST-C, so they were not included in the corresponding analysis. Sixteen subjects were excluded from the analysis due to missing information on covariates such as BMI or SES. All the excluded participants due to missing BMI or SES values were diagnosed with a current psychiatric disorder. There were no significant differences in either sociodemographic factors or cortisol values when comparing the participants excluded and subjects with psychiatric diagnostic included in the analysis; however, the excluded participants exhibited significantly higher CGAS than those included (*t* = 2.360, *p* = 0.020).

A brief summary of the sociodemographic and anthropometric variables, by CM history, is provided in Table 1. Significant group differences according to CM exposure were observed with regard to age, ethnicity, SES, illegal drug use, CGAS, GFES, BMI, current psychiatric disorder, and type of clinical care unit. Mean cortisol values by CM group measures at each diurnal and TSST-C timepoint, AUCg and AUCi values, and STAI-Trait and STAI-State scores are summarized in Table 1.

### Childhood maltreatment and diurnal salivary cortisol

**Fig. 2.**
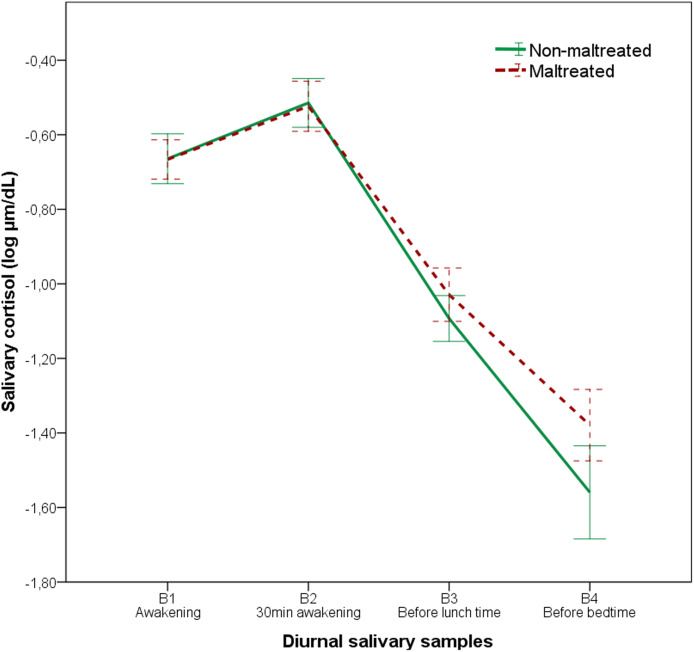
Diurnal salivary cortisol in participants with and without CM. Exposure to CM significantly increased AUCg levels, indicating a higher total diurnal cortisol output. Specifically, youth exposed to CM showed increased cortisol levels before bedtime (B4). The analysis was adjusted for sex, pubertal stage, clinical status, time of the first cortisol sample collection (B1), psychopharmacological treatment, illegal drugs use, ethnicity, corticosteroid medication, oral contraceptive use, BMI, and socioeconomic status.

As expected, cortisol levels fluctuated significantly throughout the day, following a circadian rhythm (*F* = 218.307, *p* < 0.001). No global interaction between time and CM was detected (*F* = 1.467, *p* = 0.225), reflecting a similar cortisol diurnal trajectory in both groups (see Table 2), also evidenced by AUCi levels, *F*_(1,160)_ = 3.040, *p* = 0.083, *η_p_*^2^ = 0.021. However, the simple effects analysis in the context of mixed-effect model revealed a significant time point-specific interaction at B4 (before bedtime) between CM groups (*F* = 4.678, *p* = 0.032). Although cortisol levels consistently decreased from lunchtime to bedtime in both groups, this was less pronounced in the CM group, leading to a higher total hormonal output over the whole day, as evidenced by a higher AUCg, *F*_(1,160)_ = 12.244, *p* = 0.001, *η_p_*^2^ = 0.079 (see Table 2 and Fig. 2). No significant interactions have been reported between CM and clinical status, pubertal stage, or sex. The effect of clinical status, pubertal stage, and sex on diurnal cortisol levels is reported in the online Supplementary material. Similar results were observed in the diurnal cortisol response when considering only subjects with confirmed CM (see online Supplementary material).

Neither the frequency nor the severity of CM was associated with diurnal cortisol slope during the day, *F*_severity_ = 1.214, *p* = 0.300; *F*_frequency_ = 1.085, *p* = 0.372, reflecting a similar cortisol diurnal trajectory between groups, also evidenced by AUCi, *F*_severity(2,160)_ = 1.716, *p* = 0.184, *η_p_*^2^ = 0.024; *F*_frequency(2,160)_ = 1.276, *p* = 0.282, *η_p_*^2^ = 0.018. However, the simple effect analysis revealed a significant interaction at B4 (before bedtime); participants exposed to moderate/severe CM experiences or often/frequently exposed to CM showed higher cortisol levels before bedtime when compared with subjects without CM (*p*_severity_ = 0.020; *p*_frequency_ = 0.048). The AUCg levels suggested a dose–response relationship between CM severity/frequency and total cortisol output during the day, *F*_severity(2,160)_ = 6.349, *p* = 0.002, *η_p_*^2^ = 0.084; *F*_frequency(2,160)_ = 6.068, *p* = 0.003, *η_p_*^2^ = 0.081. As expected, these results were even more significant when dichotomizing the sample according to the severity/frequency of CM as either: (1) no/low exposure or (2) moderate/severe exposure (see online Supplementary material).

### Childhood maltreatment and salivary cortisol response during acute psychosocial stress (TSST-C)

**Fig. 3.**
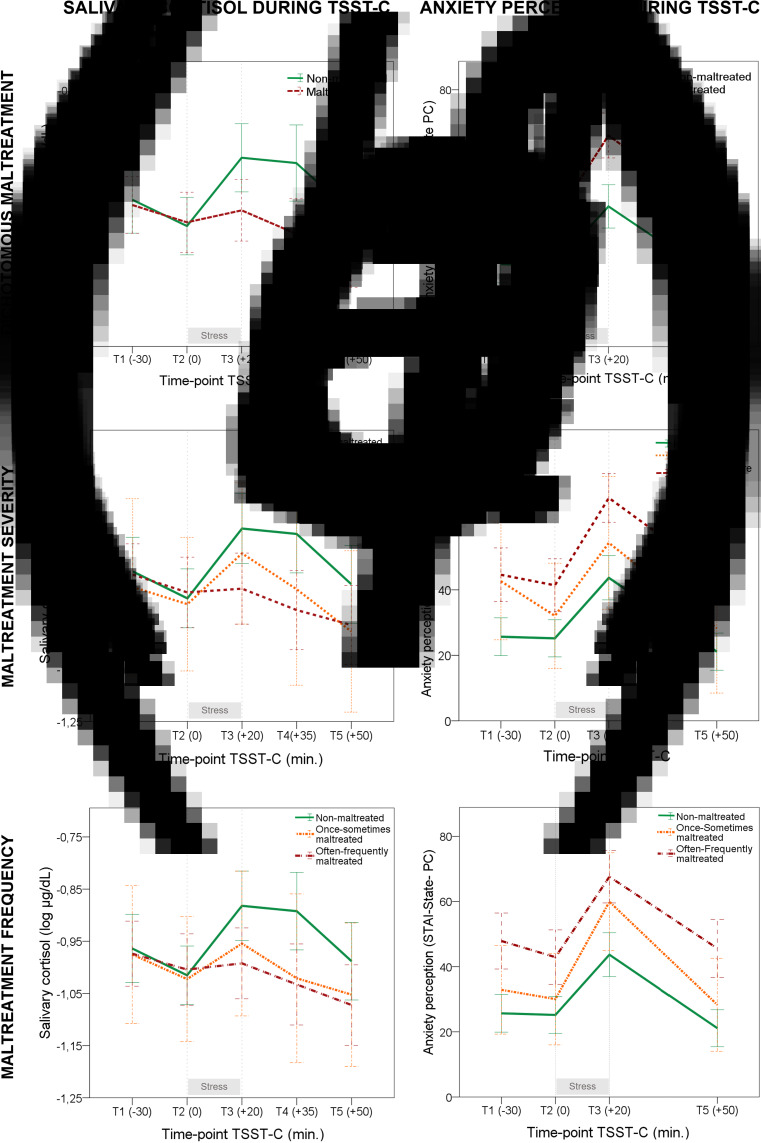
Salivary cortisol response and anxiety perception during the Trier Social Stress Test for children (TSST-C) according to CM. (*a*) Subjects without CM had increased cortisol levels after exposure to acute psychosocial stress, while in those with a history of CM the cortisol levels remained stable. (*b*) Anxiety perception increased by the same magnitude in both participants with and those without a history of CM, after exposure to psychosocial stress. However, subjects with CM showed higher overall levels of anxiety during the protocol. (*c*) Participants without CM or low exposure to CM had a similar pattern of HPA-axis response during the TSST-C, increasing cortisol levels after acute stress. However, those exposed to moderate/severe CM showed a blunted cortisol response when faced with acute psychosocial stress, indicating hyporeactivity of the HPA-axis. (*d*) Anxiety perception increased by the same magnitude in all subjects, after exposure to psychosocial stress. However, youth with CM, both with low and moderate/severe exposure, had higher overall levels of anxiety during the protocol when compared with non-maltreated participants. (*e*) Subjects without CM and those who suffered CM once/sometimes had a similar pattern of HPA-axis response during the TSST-C. However, those exposed to CM often/frequently showed lower levels of cortisol after exposure to acute psychosocial stress, indicating hyporeactivity in the HPA-axis during acute psychosocial stress. (*f*) Anxiety perception increased by the same magnitude in all the subjects after exposure to psychosocial stress. However, youth with CM, both those who suffered CM once/sometimes and those who suffered CM often/frequently, had higher overall levels of anxiety. The analysis was adjusted for sex, pubertal stage, psychopathological diagnosis, psychopharmacological treatment, illegal drugs use, ethnicity, corticosteroid medication, oral contraceptive use, BMI, and socioeconomic status.

Cortisol levels during the TSST-C significantly differed as a function of time (*F* = 8.953, *p* < 0.001), indicating the validity of this procedure to stimulate cortisol secretion in our cohort. A significant interaction between CM and time was identified (*F* = 4.530, *p* = 0.002), indicating a different trajectory of cortisol levels during the protocol between groups of CM. Specifically, the simple effects analysis in the context of mixed-effect model revealed a significant time point-specific interaction when comparing cortisol levels at T3 (immediately after the stressful situation) (*F* = 4.993; *p* = 0.027), at T4 (15 min after the stressful situation finished) (*F* = 10.404, *p* = 0.001), and at T5 (30 min after the stressful situation finished) (*F* = 4.561, *p* = 0.034). While in individuals without CM the cortisol levels increased after acute stress, there were no changes in cortisol concentration in subjects with CM (see Fig. 3*a* and Table 2). In line with this, participants with CM showed lower levels of AUCi than those without CM, *F*_(1,165)_ = 4.779, *p* = 0.030, *η_p_*^2^ = 0.031, reflecting fewer hormonal changes over time. In contrast, CM was not associated with a global difference in cortisol levels throughout the entire TSST-C procedure (*F* = 3.015, *p* = 0.084), as also indicated by AUCg, *F*_(1,165)_ = 0.091, *p* = 0.763, *η_p_*^2^ = 0.001. Similar results were observed in cortisol response during TSST-C when considering only subjects with a confirmed history of CM (see online Supplementary material). Sex, pubertal stage, and clinical status did not interact with CM, and none of these variables explained a different response pattern during the TSST-C. However, significant differences were observed in the overall cortisol levels according to pubertal stage and clinical status. Adolescents showed higher levels of cortisol (AUCg) when compared with children, and subjects with a current psychiatric diagnosis reported lower levels of cortisol (AUCg) when compared with healthy participants (further details in the online Supplementary material).

When the severity and frequency of CM were analyzed, significant interactions were again identified between CM and time (*F*_severity_ = 2.773, *p* = 0.006; *F*_frequency_ = 2.665, *p* = 0.008). Specifically, the simple effects analysis revealed a significant time point-specific interaction when comparing cortisol levels at T3 (immediately after the stressful situation) (*p*_severity_ = 0.012; *p*_frequency_ = 0.026), at T4 (15 min after the stressful situation finished) (*p*_severity_ = 0.001; *p*_frequency_ = 0.001), and at T5 (30 min after the stressful situation finished) (*p*_severity_ = 0.033; *p*_frequency_ = 0.023). While subjects without CM showed an increase in cortisol levels after the stressor, those exposed to moderate/severe or often/frequent CM were characterized by a blunted response, suggesting a dose–response relationship between CM severity/frequency and cortisol fluctuation during the TSST-C (see Fig. 3*c* and *e*). In this vein, participants exposed to moderate/severe and often/frequent CM displayed significantly lower values of AUCi than those without CM or exposed to low severity/frequency of CM, *F*_severity(2,165)_ = 3.921, *p* = 0.022, *η_p_*^2^ = 0.052; *F*_frequency(2,165)_ = 3.194, *p* = 0.044, *η_p_*^2^ = 0.042 (see Table 2). As expected, these results were even more significant when a new dichotomization was performed for severity/frequency of CM as either: (1) none or low and (2) moderate or severe exposure (for details see online Supplementary material). No significant differences in overall cortisol levels during the protocol were observed between severity/frequency groups of CM (*F*_severity_ = 1.736, *p* = 0.179; *F*_frequency_ = 1.839, *p* = 0.162), also evidenced by AUCg, *F*_severity(2, 165)_ = 0.057, *p* = 0.945, *η_p_*^2^ = 0.001; *F*_frequency(2, 165)_ = 0.074, *p* = 0.929, *η_p_*^2^ = 0.001.

### Childhood maltreatment, anxiety trait, and anxiety perception during acute psychosocial stress (TSST-C)

Participants with CM exhibited significantly higher levels of anxiety trait than those without CM, *F*_(1,160)_ = 9.129, *p* = 0.003, *η_p_*^2^ = 0.060. The severity and frequency of CM were also associated with anxiety trait, *F*_severity(2,160)_ = 5.109, *p* = 0.007, *η_p_*^2^ = 0.062; *F*_frequency(2,160)_ = 4.102, *p* = 0.019, *η_p_*^2^ = 0.056, with the lowest anxiety trait levels exhibited by subjects none exposed to CM (see Table 2). No significant correlation between anxiety trait and overall diurnal cortisol levels was found (see online Supplementary material).

As seen in Fig. 3*b*, the TSST-C consistently increased perceived anxiety after acute stress in all the subjects (*F* = 34.544, *p* < 0.001). However, there were no interactions between time and CM (*F* = 1.742, *p* = 0.160), reflecting similar trajectories of perceived anxiety during the acute psychosocial stress in both subjects with and those without CM. Furthermore, those with CM showed higher overall perceived anxiety during the entire procedure than subjects without CM (*F* = 23.836, *p* < 0.001). Moreover, in youth without CM, anxiety perception during the TSST-C was negatively correlated with cortisol levels, but not in youth exposed to CM (see online Supplementary material). Subjects exposed to both low and high severity/frequency of CM showed higher overall levels of anxiety during the whole protocol than subjects without CM, *F*_severity_ = 11.112, *p* *<* 0.001; *F*_frequency_ = 12.142, *p* < 0.001 (see Fig. 3*c* and *d*). However, there were no differences between groups in the magnitude of the increase of perceived anxiety after the acute stressor, *F*_severity_ = 1.670, *p* = 0.131; *F*_frequency_ = 1.240, *p* = 0.287, *η_p_*^2^ = 0.022, with all groups exhibiting the same trajectory (see Table 2). Similar results were obtained when considering only subjects with a confirmed history of CM (for details see online Supplementary material).

## Discussion

The present study elucidated how the proximal CM in children and adolescents impacts on HPA-axis functioning and on anxiety perception. In summary, youth exposed to CM, regardless of the presence of a current psychopathology, showed (i) a basal disruption of the HPA-axis circadian rhythm with increased daily cortisol levels, (ii) reduced HPA-axis reactivity during an acute psychosocial stress, and (iii) increased anxiety perception as a trait and during the whole psychosocial stress episode. Interestingly, all the subjects exposed to CM experienced heightened anxiety but only those exposed to more severe or frequent CM exhibited significant HPA-axis dysregulation. To the best of our knowledge, this is the first study to date to report the impact of CM severity measured as the gravity of the experiences suffered, rather than as the accumulation of different types of CM (e.g. pinch with momentary redness considered as low physical abuse, *v.* physical aggression that needs medical intervention considered as very severe).

Our results suggest that subjects who have suffered CM have higher overall diurnal cortisol levels. Specifically, the participants with CM were characterized by a blunted decline of cortisol levels from lunchtime to bedtime, compared with those without CM. This alteration of the circadian cortisol rhythm is consistent with the presence of hypercortisolism, as evidenced by higher AUCg scores in the group exposed to CM, especially those exposed to more severe and frequent CM. This may indicate a desynchrony trend in this intrinsic biological process, which has been described as a risk factor for rising mental health symptoms. Our findings are accordant with other studies focused on CM, which have reported both a blunted decline in HPA-axis activity throughout the day (Bernard, Zwerling, & Dozier, 2015) and higher overall cortisol output (Cicchetti & Rogosch, 2001). Our results could help to elucidate the co-occurrence of hypercortisolism and a flattened diurnal cortisol response, as high diurnal cortisol levels may be explained by an atypical diurnal decline. Similar findings have been reported in adults exposed to childhood adversities, suggesting the persistence of a less pronounced diurnal cortisol slope (Kuras et al., 2017). This HPA-axis dysregulation has important implications for other biological functions, as immune system (e.g. compromising the release of pro and anti-inflammatory substances) ultimately contributes to the increased risk of chronic disease later in life.

Although a recent meta-analysis (Bernard et al., 2017) reported no overall effect of CM on the diurnal cortisol slope, the authors also discussed the impact of many confounders. For example, age may influence the association between CM and cortisol rhythms; whereas cortisol levels could be elevated soon after the onset of a stressor (hypercortisolism), they could decrease over time, reflecting a pattern of hypocortisolism in adulthood (Miller, Chen, & Zhou, 2007). Although we did not observe this interaction between CM and pubertal stage, diurnal cortisol levels showed to be higher in adolescents when compared to children. Furthermore, our findings suggest that CM is associated with biological alterations also in youth without psychiatric disorders. In this regard, different approaches suggest that resilient subjects, who were exposed to CM but are asymptomatic, may present a particular neurobiological adaptive response, as brain connectivity changes to compensate for the alterations caused by abuse (Ohashi et al., 2019).

Secondly, regarding the HPA-axis response to acute psychosocial stress, consistent with the extant literature (Bunea et al., 2017), children and adolescents exposed to CM exhibited a blunted cortisol response during the TSST-C, compared with those without CM. While previous literature supports that the blunted cortisol response is better observed in adult populations (while arguing that smaller effects are seen in children and adolescents due to HPA-axis hyperactivation following immediate trauma), an early hypoactivation is already observed in our sample, as has been reported previously (MacMillan et al., 2009). Although subjects exposed to CM remained hyporeactive under acute stress, in terms of HPA-axis activity, they experienced a significant increase in perceived anxiety, equivalent to that experienced by those not exposed to CM. This reveals a clear dissociation between anxiety perception and the physiological response to stressful situations in young people with CM, which might impair their ability to manage appropriately and cope with everyday emotionally negative situations (Liu et al., 2012). Notably, emotion regulation deficits have been suggested as a key pathway linking CM with psychopathology (Dvir, Ford, Hill, & Frazier, 2014; Hart et al., 2018). Further studies are required to explore which biomarkers other than cortisol might be linked with heightened anxiety in subjects exposed to CM (Quidé et al., 2019). Our results further suggest that, although participants with a current psychopathology tended to have lower cortisol levels in general, the HPA-axis alterations in subjects exposed to CM were present in both subjects with and without a current psychopathology. Contrary to some previous findings, in our sample neither pubertal stage (Gunnar, Wewerka, Frenn, Long, & Griggs, 2009) nor sex (Trickett et al., 2014) interacted with CM to predict HPA-axis reactivity during the TSST-C.

Furthermore, besides the impact of CM on HPA-axis activity and increased levels of anxiety (trait and state), our findings also showed that the severity and frequency of CM play a key role, thereby supporting a dose–response relationship (Anda et al., 2006). Thus, in line with Trickett et al. (2014), subjects exposed to more severe or more frequent forms of CM manifested the most subdued HPA-axis responses under basal conditions and in response to psychosocial stress; notably, Trickett et al. considered severity as the accumulation of different types of CM, rather than according to the specific characteristics of the experiences suffered. These findings warn of the deleterious impact that milder forms of CM may have once they become chronic. This is important as children who experience mild CM are often not detected or receive less clinical and social care (Humphreys, 2020). Furthermore, it seems that these children start showing higher levels of perceived anxiety before there is a marked biological dysregulation, offering a window of opportunity for early detection and intervention. Hence, the use of accurate child screening instruments at subclinical stages should be generalized, since most children are only identified once they already have severe psychiatric symptomatology (Bailhache, Leroy, Pillet, & Salmi, 2013). Moreover, since dysfunction in neurobiological systems negatively impacts treatment outcomes, youth with CM may also require specific treatment adapted to their condition (Tyrka, Burgers, Philip, Price, & Carpenter, 2013).

The methodology used in the present study includes a wide range of CM experiences reported from different sources, since there is often a substantial gap between subjects identified in informant-based studies and self-report assessments (Baldwin, Reuben, Newbury, & Danese, 2019). Thus, our findings suggest that participants with a suspected history of CM identified by clinicians show the same HPA-axis dysfunctions as subjects with a confirmed history of CM. Likewise, given that CM studies may lack sensitivity when the experiences are not qualitatively assessed (via the severity and frequency of exposure), key information may be lost and findings distorted. This highlights the need for specific training of clinicians in child psychiatric and pediatric services, so CM assessment can be routinely implemented, despite the time and effort required to perform such complex assessments (Zeanah & Edm, 2018).

Although prior evidence suggests that exposure to CM during middle childhood has the greatest effects on emotional dysregulation (Dunn, Nishimi, Gomez, Powers, & Bradley, 2018), it is difficult to pinpoint the exact developmental period when HPA-axis functioning is disrupted. Future research should incorporate more detailed information about the timing and proximity of CM to delineate vulnerable periods (Andersen & Teicher, 2008). It would be interesting to study the clinical course of the children to identify possible risk and protective factors for the future onset of psychopathology. A more dimensional approach focused on symptom dimensions might reveal varying patterns of adrenocortical regulation (Cicchetti & Rogosch, 2001). It is important to note that CM is not a phenomenon that can be studied in isolation, since both its causes and consequences are systematic and there are many factors that must be taken into account in order to fully understand it.

The blunted reactivity observed in our study supports plausible habituation, i.e. chronic exposure to stress may be linked with an adaptive desensitization to new stressors over time (Murali & Chen, 2005). These latent neurobiological alterations could drive an increased vulnerability to psychopathology during childhood and adolescence (Busso et al., 2017), which may persist, leading to the onset of a wide range of psychiatric conditions in adulthood (Kudielka & Wüst, 2010). Other factors with the potential to moderate the consequences of CM should also be taken into account, such as the type of CM suffered, the relationship with the abuser, social support received, and coexistence of other types of trauma such as bullying (Arseneault, 2018), domestic violence (Osofsky, 2018), or recent stressful life events (March-Llanes, Marqués-Feixa, Mezquita, Fañanás, & Moya-Higueras, 2017).

One of the limitations of the current study is the methodology used for assessing the presence and characteristics of CM exposure. Widely used questionnaires such as the CTQ cannot be administered to children younger than 12 years; indeed, there is no validated questionnaire to assess the presence of CM in the 7–17 years range. The main reason behind this is that younger children have a limited understanding of their own exposure, since they are still cognitively immature. Thus, any assessment of CM in this vulnerable population needs to be adjusted to maximize the reliable information that can be captured from the different informants (not only the child) and, at the same time, to minimize the trauma that the interview itself can represent to a victimized child. Thus, use of TASSCV allows the proper assessment of children and adolescents exposed from milder to severe forms of CM, which would have otherwise not been identified. Unfortunately, use of TASSCV requires a longer time for a proper assessment together with the gathering of information from multiple informants, which might make it more challenging to use than simply relying on short self-administered questionnaires such as the CTQ or considering only the most severe children already detected by social services. Since most of the sample was recruited in psychiatric units, there is an unusually high proportion of ADHD cases in the non-CM group; thus, our findings might not be generalizable to other populations. At the same time, the majority of CM-exposed subjects suffered from some sort of psychiatric condition, while most participants non-exposed to CM had no psychopathological history. Further research including a higher proportion of subjects exposed to CM with no psychiatric symptomatology (i.e. resilient) is required to disentangle the role of CM in the development of HPA-axis disturbances and whether the later precede the onset of psychiatric disorders.

## Conclusions

CM affects multiple domains of life such as intimate relationships, violence and criminal offending, employment, drug abuse, and physical and mental health (Hughes et al., 2017). It is a serious global health problem with staggering long-term economic costs (Thielen et al., 2016). This study is intended to raise awareness of the biological and clinical repercussions of CM during or proximally to exposure, encouraging clinicians to ask patients about CM history and to respond accordingly, seeking therapeutic alternatives to manage acute stress better. Children exposed to CM and attended in child protection units, child psychiatric, or pediatric units are still at a sensitive period of neurological, cognitive, social, and emotional development, during which high-quality interventions can make an important difference and shift the balance between risk and protective factors (Chinitz, Guzman, Amstutz, Kohchi, & Alkon, 2017). Thus, family psychotherapeutic interventions have the potential to normalize HPA-axis function if implemented promptly (Gunnar, DePasquale, Reid, Donzella, & Miller, 2019).

## Supplementary material

For supplementary material accompanying this paper visit https://doi.org/10.1017/S003329172100249X. click here to view supplementary material

## EPI-Young Stress GROUP

María Martín^9^, Eulalia Anglada^12^, Pilar Santamarina^2,3,4^, Patricia Rubio^6^, Iría Méndez^2,3,4^, Dolores Moreno^2,6^, María Ramos^2,6^, Jorge Vidal^2,6^, Juanjo Carballo^2,6^, Elena Font^2,3,4^, Lydia Gayubo^11^, Laura Colino^11^, María Rodrigo-Yanguas^11^, Maddi Laborde^8^, Jaume March-Llanes^5^.

## References

[ref1] Allen, A. P., Kennedy, P. J., Cryan, J. F., Dinan, T. G., & Clarke, G. (2014). Biological and psychological markers of stress in humans: Focus on the Trier Social Stress Test. Neuroscience and Biobehavioral Reviews, 38, 94–124. 10.1016/j.neubiorev.2013.11.005. 24239854

[ref2] Anda, R. F., Felitti, V. J., Bremner, J. D., Walker, J. D., Whitfield, C., Perry, B. D., … Giles, W. H. (2006). The enduring effects of abuse and related adverse experiences in childhood: A convergence of evidence from neurobiology and epidemiology. European Archives of Psychiatry and Clinical Neuroscience, 256(3), 174–186. 10.1007/s00406-005-0624-4. 16311898 PMC3232061

[ref3] Andersen, S. L., & Teicher, M. H. (2008). Stress, sensitive periods and maturational events in adolescent depression. Trends in Neuroscience, 31(4), 183–191. 10.1016/j.tins.2008.01.004. 18329735

[ref4] APA: American Psychiatric Association. (2013). DSM-5: Diagnostic and statistical manual of mental disorders (5th ed.). Washington, DC: American Psychiatric Publishing.

[ref5] Arseneault, L. (2018). Annual Research Review: The persistent and pervasive impact of being bullied in childhood and adolescence: Implications for policy and practice. Journal of Child Psychology and Psychiatry, and Allied Disciplines, 59(4), 405–421. 10.1111/jcpp.12841. 29134659 PMC6542665

[ref6] Bailhache, M., Leroy, V., Pillet, P., & Salmi, L.-R. (2013). Is early detection of abused children possible?: A systematic review of the diagnostic accuracy of the identification of abused children. BMC Pediatrics, 13(1), 1–11. 10.1186/1471-2431-13-202. 24314318 PMC4029314

[ref7] Baldwin, J. R., Reuben, A., Newbury, J. B., & Danese, A. (2019). Agreement between prospective and retrospective measures of childhood maltreatment: A systematic review and meta-analysis. JAMA Psychiatry, 76(6), 584–593. 10.1001/jamapsychiatry.2019.0097. 30892562 PMC6551848

[ref8] Bernard, K., Frost, A., Bennett, C. B., & Lindhiem, O. (2017). Maltreatment and diurnal cortisol regulation: A meta-analysis. Psychoneuroendocrinology, 78, 57–67. 10.1016/j.psyneuen.2017.01.005. 28167370

[ref9] Bernard, K., Zwerling, J., & Dozier, M. (2015). Effects of early adversity on young children’ s diurnal cortisol rhythms and externalizing behavior. Developmental Psychobiology, 57(8), 935–947. 10.1002/dev.21324. 26289841 PMC5773098

[ref10] Bernstein, D. P., Stein, J. A., Newcomb, M. D., Walker, E., Pogge, D., Ahluvalia, T., … Zule, W. (2003). Development and validation of a brief screening version of the Childhood Trauma Questionnaire. Child Abuse and Neglect, 27(2), 169–190. 10.1016/S0145-2134(02)00541-0. 12615092

[ref11] Brown, G. W., Harris, T. O., & Craig, T. K. J. (2019). Exploration of the influence of insecure attachment and parental maltreatment on the incidence and course of adult clinical depression. Psychological Medicine, 49, 1025–1032. 10.1017/S0033291718001721. 30107862

[ref12] Bunea, I. M., Szentágotai-t, A., & Miu, A. C. (2017). Early-life adversity and cortisol response to social stress: A meta-analysis. Translational Psychiatry, 7(12), 1–8. 10.1038/s41398-017-0032-3. PMC5802499 29225338

[ref13] Buske-Kirschbaum, A., Jobst, S., Wustmans, A., Kirschbaum, C., Rauh, W., & Hellhammer, D. (1997). Attenuated free cortisol response to psychosocial stress in children with atopic dermatitis. Psychosomatic Medicine, 59(4), 419–426. 10.1097/00006842-199707000-00012. 9251162

[ref14] Busso, D. S., Mclaughlin, K. A., Brueck, S., Peverill, M., Gold, A. L., & Sheridan, M. A. (2017). Child abuse, neural structure, and adolescent psychopathology: A longitudinal study. Journal of the American Academy of Child & Adolescent Psychiatry, 56(4), 321–328.e1. 10.1016/j.jaac.2017.01.013. 28335876 PMC5367472

[ref15] CARM (2012). Instrumento para la valoración de la gravedad de las situaciones de desprotección infantil (Tool for assessing the severity of situations in which children are vulnerable- TASSCV). Servicios Sociales de Atención Primaria y Especializados de La Región de Muricia. https://www.carm.es/web/pagina?IDCONTENIDO=9415&IDTIPO=246&RASTRO=c886$m5855.

[ref16] Chinitz, S., Guzman, H., Amstutz, E., Kohchi, J., & Alkon, M. (2017). Improving outcomes for babies and toddlers in child welfare: A model for infant mental health intervention and collaboration. Child Abuse and Neglect, 70(May), 190–198. 10.1016/j.chiabu.2017.05.015. 28622589

[ref17] Cicchetti, D., & Rogosch, F. A. (2001). The impact of child maltreatment and psychopathology on neuroendocrine functioning. Development and Psychopathology, 13(4), 783–804. 11771908

[ref18] de la Peña, F. R., Villavicencio, L. R., Palacio, J. D., Félix, F. J., Larraguibel, M., Viola, L., … Ulloa, R. E. (2018). Validity and reliability of the kiddie schedule for affective disorders and schizophrenia present and lifetime version DSM-5 (K-SADS-PL-5) Spanish version. BMC Psychiatry, 18(1), 1–7. 10.1186/s12888-018-1773-0. 29898698 PMC6001018

[ref19] DePasquale, C. E., Donzella, B., & Gunnar, M. R. (2019). Pubertal recalibration of cortisol reactivity following early life stress: A cross-sectional analysis. Journal of Child Psychology and Psychiatry and Allied Disciplines, 60(5), 566–575. 10.1111/jcpp.12992. 30357830 PMC6458083

[ref20] De Punder, K., Heim, C., & Entringer, S. (2019). Psychoneuroendocrinology association between chronotype and body mass index : The role of C-reactive protein and the cortisol response to stress. Psychoneuroendocrinology, 109(February), 104388. 10.1016/j.psyneuen.2019.104388. 31398588

[ref21] Dunn, E. C., Nishimi, K., Gomez, S. H., Powers, A., & Bradley, B. (2018). Developmental timing of trauma exposure and emotion dysregulation in adulthood: Are there sensitive periods when trauma is most harmful? Journal of Affective Disorders, 227(October 2017), 869–877. 10.1016/j.jad.2017.10.045. 29254068 PMC5805641

[ref22] Dvir, Y., Ford, J. D., Hill, M., & Frazier, J. A. (2014). Childhood maltreatment, emotional dysregulation, and psychiatric comorbidities. Harvard Review of Psychiatry, 22(3), 149–161. 10.1097/HRP.0000000000000014. 24704784 PMC4091823

[ref23] Fogelman, N., & Canli, T. (2018). Early life stress and cortisol: A meta-analysis. Hormones and Behavior, 98(December 2017), 63–76. 10.1016/j.yhbeh.2017.12.014. 29289660

[ref24] Forti, M. Di, Quattrone, D., Freeman, T. P., Tripoli, G., Gayer-anderson, C., Quigley, H.. (2019). The contribution of cannabis use to variation in the incidence of psychotic disorder across Europe (EU-GEI): A multicentre case-control study. The Lancet Psychiatry, 6(5), 427–436. 10.1016/S2215-0366(19)30048-3. 30902669 PMC7646282

[ref25] Gunnar, M. R., DePasquale, C. E., Reid, B. M., Donzella, B., & Miller, B. S. (2019). Pubertal stress recalibration reverses the effects of early life stress in postinstitutionalized children. Proceedings of the National Academy of Sciences, 116(48), 23984–23988. 10.1073/pnas.1909699116. PMC6883847 31712449

[ref26] Gunnar, M. R., Wewerka, S., Frenn, K., Long, J., & Griggs, C. (2009). Developmental changes in hypothalamus–pituitary–adrenal activity over the transition to adolescence: Normative changes and associations with puberty. Development and Psychopathology, 21(1), 69–85. 10.1017/S0954579409000054.Developmental. 19144223 PMC3933029

[ref27] Hart, H., Lim, L., Mehta, M. A., Simmons, A., Mirza, K. A. H., & Rubia, K. (2018). Altered fear processing in adolescents with a history of severe childhood maltreatment: An fMRI study. Psychological Medicine, 48(7), 1092–1101. 10.1017/S0033291716003585. 29429419 PMC6088776

[ref28] Hollingshead, A. B. (1975). Four factor index of social status. New Haven, CT: Yale University Department of Psychology.

[ref29] Hughes, K., Bellis, M. A., Hardcastle, K. A., Sethi, D., Butchart, A., Mikton, C., … Dunne, M. P. (2017). The effect of multiple adverse childhood experiences on health: A systematic review and meta-analysis. The Lancet Public Health, 2(8), e356–e366. 10.1016/S2468-2667(17)30118-4. 29253477

[ref30] Humphreys, K. L. (2020). Child maltreatment recurrence points to urgent need to improve systems for identification and prevention. Journal of the American Academy of Child & Adolescent Psychiatry. 10.1016/j.jaac.2020.07.005. 32710936

[ref31] Hunter, A. L., Minnis, H., & Wilson, P. (2011). Altered stress responses in children exposed to early adversity: A systematic review of salivary cortisol studies. Stress (Amsterdam, The Netherlands), 14(6), 614–626. 10.3109/10253890.2011.577848. 21675865

[ref32] Kaess, M., Parzer, P., Mattern, M., Resch, F., Bifulco, A., & Brunner, R. (2011). Childhood Experiences of Care and Abuse (CECA) – validation of the German version of the questionnaire and interview, and results of an investigation of correlations between adverse childhood experiences and suicidal behaviour. Zeitschrift fur Kinder- und Jugendpsychiatrie und Psychotherapie, 39(4), 243–252. 10.1024/1422-4917/a000115. 21667449

[ref33] King, L. S., Colich, N. L., LeMoult, J., Humphreys, K. L., Ordaz, S. J., Price, A. N., & Gotlib, I. H. (2017). The impact of the severity of early life stress on diurnal cortisol: The role of puberty. Psychoneuroendocrinology, 77, 68–74. 10.1016/j.psyneuen.2016.11.024. 28024271 PMC5336485

[ref34] Koss, K. J., & Gunnar, M. R. (2018). Annual Research Review : Early adversity, the hypothalamic – pituitary – adrenocortical axis, and child psychopathology. Journal of Child Psychology and Psychiatry, 59(4), 327–346. 10.1111/jcpp.12784. 28714126 PMC5771995

[ref35] Kudielka, B. M., & Wüst, S.. (2010). Human models in acute and chronic stress: Assessing determinants of individual hypothalamus–pituitary–adrenal axis activity and reactivity. Stress, 13(1), 1–14. 10.3109/10253890902874913. 20105052

[ref36] Kuras, Y. I., Assaf, N., Thoma, M. V., Gianferante, D., Hanlin, L., Chen, X., … Rohleder, N. (2017). Blunted Diurnal Cortisol Activity in Healthy Adults with Childhood Adversity, 11(November), 1–8. 10.3389/fnhum.2017.00574. PMC5712303 29234280

[ref37] Lê-scherban, F., Brenner, A. B., Hicken, M. T., Needhamm, B. L., Seeman, T., Sloan, R. P., … Roux, A. V. D. (2018). Child and adult socioeconomic status and the cortisol response to acute stress: Evidence from the Multi-Ethnic Study of Atherosclerosis. Psychosomatic Medicine, 80(2), 184–192. 10.1097/PSY.0000000000000543. 29215456 PMC5794563

[ref38] Lippard, E. T. C., & Nemeroff, C. B.. (2020). The devastating clinical consequences of child abuse and neglect: Increased disease vulnerability and poor treatment response in mood disorders. American Journal of Psychiatry, 177(1), 20–36. 10.1176/appi.ajp.2019.19010020. 31537091 PMC6939135

[ref39] Liu, J., Chaplin, T. M., Wang, F., Sinha, R., Mayes, L. C., & Blumberg, H. P. (2012). Stress reactivity and corticolimbic response to emotional faces in adolescents. JAAC, 51(3), 304–312. 10.1016/j.jaac.2011.12.014. PMC3292764 22365466

[ref40] MacMillan, H. L., Georgiades, K., Duku, E. K., Shea, A., Steiner, M., Niec, A., … Schmidt, L. A. (2009). Cortisol response to stress in female youths exposed to childhood maltreatment: Results of the youth mood project. Biological Psychiatry, 66(1), 62–68. 10.1016/j.biopsych.2008.12.014. 19217075 PMC3816014

[ref41] Marceau, K., & Abel, E. (2018). Mechanisms of cortisol – substance use development associations: Hypothesis generation through gene enrichment analysis. Neuroscience and Biobehavioral Reviews, 92, 128–139. 10.1016/j.neubiorev.2018.05.020. 29802855 PMC6310615

[ref42] March-Llanes, J., Marqués-Feixa, L., Mezquita, L., Fañanás, L., & Moya-Higueras, J. (2017). Stressful life events during adolescence and risk for externalizing and internalizing psychopathology: A meta-analysis. European Child and Adolescent Psychiatry, 26(12), 1409–1422. 10.1007/s00787-017-0996-9. 28502034

[ref43] Miller, G. E., Chen, E., & Zhou, E. S. (2007). If it goes up, must it come down? Chronic stress and the hypothalamic-pituitary-adrenocortical axis in humans. Psychological Bulletin, 133, 25–45. 10.1037/0033-2909.133.1.25. 17201569

[ref44] Morris, N. M., & Udry, J. R. (1980). Validation of a self-administered instrument to assess stage of adolescent development. Journal of Youth and Adolescence, 9(3), 271–280. 10.1007/BF02088471. 24318082

[ref45] Murali, R., & Chen, E. (2005). Exposure to violence and cardiovascular and neuroendocrine measures in adolescents. Annals of Behavioral Medicine, 30(2), 155–163. 16173912 10.1207/s15324796abm3002_8

[ref46] Ohashi, K., Anderson, C. M., Bolger, E. A., Khan, A., McGreenery, C. E., & Teicher, M. H. (2019). Susceptibility or resilience to maltreatment can be explained by specific differences in brain network architecture. Biological Psychiatry, 85(8), 690–702. 10.1016/j.biopsych.2018.10.016. 30528381 PMC6440838

[ref47] Osofsky, J. D. (2018). Commentary: Understanding the impact of domestic violence on children, recognizing strengths, and promoting resilience: Reflections on Harold and Sellers (2018). Journal of Child Psychology and Psychiatry, and Allied Disciplines, 59(4), 403–404. 10.1111/jcpp.12902. 29574733

[ref48] Ouellet-Morin, I., Robitaille, M. P., Langevin, S., Cantave, C., Brendgen, M., & Lupien, S. J. (2019). Enduring effect of childhood maltreatment on cortisol and heart rate responses to stress: The moderating role of severity of experiences. Development and Psychopathology, 31(2), 497–508. 10.1017/S0954579418000123. 29606171

[ref49] Provençal, N., Arloth, J., Cattaneo, A., Anacker, C., & Cattane, N. (2019). Glucocorticoid exposure during hippocampal neurogenesis primes future stress response by inducing changes in DNA methylation. Proceedings of the National Academy of Sciences, 117(38), 23280–23285. 10.1073/pnas.1820842116. PMC7519233 31399550

[ref50] Pruessner, J. C., Kirschbaum, C., Meinlschmidt, G., & Hellhammer, D. H. (2003). Two formulas for computation of the area under the curve represent measures of total hormone concentration versus time-dependent change. Psychoneuroendocrinology, 28(4), 916–931. 10.1016/j.psyneuen.2003.10.002. 12892658

[ref51] Quidé, Y., Bortolasci, C. C., Spolding, B., Kidnapillai, S., Watkeys, O. J., Cohen-Woods, S., … Green, M. J. (2019). Association between childhood trauma exposure and pro-inflammatory cytokines in schizophrenia and bipolar-I disorder. Psychological Medicine, 49(16), 2736–2744. 10.1017/S0033291718003690. 30560764

[ref52] Rey, J. M., Singh, M., Hung, S., Dossetor, D. R., Newman, L., Plapp, J. M., & Bird, K. D. (1997). A global scale to measure the quality of the family environment. Archives of General Psychiatry, 54(9), 817–822. 10.1001/archpsyc.1997.01830210061006. 9294372

[ref53] Shaffer, D., Gould, M. S., Brasic, J., Ambrosini, P., Fisher, P., Bird, H., & Aluwahlia, S. (1983). A Children's Global Assessment Scale (CGAS). Archives of General Psychiatry, 40(11), 1228–1231. 10.1001/archpsyc.1983.01790100074010. 6639293

[ref54] Spielberger, C. D. (1973). Inventario de Ansiedad Estado – Rasgo para niños, STAIC. Palo alto, CA: Consulting Psychologists Press.

[ref55] Tarullo, A. R., & Gunnar, M. R. (2006). Child maltreatment and the developing HPA axis. Hormones and Behavior, 50(4), 632–639. 10.1016/j.yhbeh.2006.06.010. 16876168

[ref56] Thielen, F. W., ten Have, M., de Graaf, R., Cuijpers, P., Beekman, A., Evers, S., & Smit, F. (2016). Long-term economic consequences of child maltreatment: A population-based study. European Child and Adolescent Psychiatry, 25(12), 1297–1305. 10.1007/s00787-016-0850-5. 27154047

[ref57] Trickett, P. K., Gordis, E., Peckins, M. K., & Susman, E. J. (2014). Stress reactivity in maltreated and comparison male and female young adolescents. Child Maltreatment, 19(1), 27–37. 10.1177/1077559513520466. 24482544

[ref58] Turner, A. I., Smyth, N., Hall, S. J., Torres, S. J., Hussein, M., Jayasinghe, S. U., … Clow, A. J. (2020). Psychological stress reactivity and future health and disease outcomes: A systematic review of prospective evidence. Psychoneuroendocrinology, 114(January), 104599. 10.1016/j.psyneuen.2020.104599. 32045797

[ref59] Tyrka, A. R., Burgers, D. E., Philip, N. S., Price, L. H., & Carpenter, L. L. (2013). The neurobiological correlates of childhood adversity and implications for treatment. Acta Psychiatrica Scandinavica, 128(6), 434–447. 10.1111/acps.12143. 23662634 PMC4467688

[ref60] Vachon, D. D., Krueger, R. F., Rogosch, F. A., & Cicchetti, D. (2015). Assessment of the harmful psychiatric and behavioral effects of different forms of child maltreatment. JAMA Psychiatry, 72(11), 1135–1142. 10.1001/jamapsychiatry.2015.1792. 26465073 PMC4699442

[ref61] Zeanah, C. H., & Edm, K. L. H. (2018). Child abuse and neglect. Journal of the American Academy of Child & Adolescent Psychiatry, 57(9), 637–644. 10.1016/j.jaac.2018.06.007. 30196867 PMC6615750

