# Supplementary material for: Childhood maltreatment disrupts HPA-axis activity under basal and stress conditions in a dose–response relationship in children and adolescents
Source: Psychol Med. 2021 Jul 16;53(3):1060–73. doi: 10.1017/S003329172100249X (PMC9976019; doi:10.1017/S003329172100249X)
Supplement: Supplementary file 1 [file S003329172100249Xsup001.docx]

**SUPPLEMENTARY MATERIAL**

1. Supplementary methods
   1. Tool for assessing the severity of situations in which children are vulnerable (TASSCV)

- Table S1: Example to assess physical needs (food) of physical neglect by TASSCV criteria
  1. *Ad-hoc* hetero-administered questionnaire of maltreatment for children under 12.
- Table S2: Items on the *ad-hoc* hetero-administered questionnaire to assess the maltreatment of children under 12
  1. Trier Social Stress Test for children (TSST-C)
  2. Salivary cortisol determination
- Table S3: Percentage of salivary cortisol samples with a coefficient of variation (CV%) higher than 30% included in the final analysis

1. Supplementary results
   1. Sensitivity analysis including only subjects with confirmed history of CM
      1. Diurnal salivary cortisol
      2. Salivary cortisol response during the TSST-C
      3. Anxiety levels during the TSST-C
   2. The effect of a current psychopathology, pubertal stage and sex on cortisol levels in confirmed and suspected CM subjects
      1. Diurnal salivary cortisol

- Figure S1: Diurnal cortisol levels in children and adolescents with and without current psychiatric disorder
  - 1. Salivary cortisol response during the TSST-C
- Figure S2: Cortisol levels during TSST-C in children and adolescents
  - 1. Anxiety levels during the TSST-C
  1. Sensitivity analysis classifying the groups as low/high CM depending on severity/frequency
     1. Diurnal salivary cortisol
     2. Cortisol reactivity during the TSST-C
  2. Relationship between anxiety perception and cortisol secretion in confirmed and suspected CM subjects
     1. Diurnal salivary cortisol
     2. Cortisol reactivity during the TSST-C
- Figure S3: Relation between anxiety perception and cortisol secretion during the TSST-C

1. Supplementary Histograms

- Figures S4: Histograms of age and pubertal state
- Figures S5: Histograms of diurnal cortisol concentration
- Figures S6: Histograms of cortisol concentration during TSST-C

4. References

**1. Supplementary methods**

**1.1. Tool for assessing the severity of situations in which children are vulnerable (TASSCV)**

The *EPI_young_stress* research project assessed situations involving maltreatment of children that the participants in the study were found to be in, using the instrument "Tool for assessing the severity of situations in which children are vulnerable” (TASSCV) which has been validated by professionals working in child and adolescent care in Spain (CARM, 2012). Specifically, the instrument was created, revised and validated over a 2-year period within the framework of the experimental programme "Detection, Notification and Registration of Cases of Maltreatment of Children in the Region of Murcia" carried out on behalf of the Directorate-General for Social Affairs, Equality and Immigration in collaboration with the Ministry of Health, Social Policy and Equality.

As explained in the original manual, this instrument forms part of a set of activities, documents and tools developed by different teams of professionals that has made it possible to establish technical criteria that allow professionals: i) to identify accurately the type of child vulnerability within the child’s family context and, ii) to assess the severity of the vulnerability. The instrument was also designed to promote greater agility and effectiveness among child care services, thereby making it easier for professionals to collaborate. To this end, we studied the use of this instrument to facilitate the detection of new cases during the examination of the participants in the study. This means that it will be possible to extrapolate the results of the study to the everyday experiences of professionals working in child care services where they can be put to practical use.

Specifically, the situations involving child vulnerability that the instrument evaluates are:

1. **Physical neglect**
   1. In terms of physical needs:
      - Food
      - Physical health
      - Clothes
      - Personal hygiene
      - Hygienic conditions at home
      - Stability and living conditions at home
   2. In terms of safety:
      - Supervision and risk prevention
      - Protection against abuse perpetrated by third parties
   3. In terms of cognitive needs
2. **Emotional neglect**
   1. In terms of interaction and affection
   2. In terms of specific attention to emotional problems
   3. In terms of rules, limits and positive values
3. **Emotional abuse**
4. **Physical abuse**
   1. Physical aggression
   2. Being given alcohol or drugs
   3. Deliberate deprivation of food or water
   4. Confinement or physical restraint
5. **Sexual abuse**
6. **Sexual exploitation**
7. **Labour exploitation**
8. **Being introduced into crime**
9. **Munchausen syndrome**
10. **Prenatal abuse**
11. **Unsuitable life models**
12. **Others**
    1. Parents unable to control the child's behaviour
    2. Abandonment
    3. Renunciation
    4. Begging

Given the huge degree of complexity involved in assessing maltreatment of children, the reference clinicians in the *EPI_young_stress* study assessed all these areas after conducting clinical interviews with the participating families and children, completing the validated maltreatment questionnaires (cited in previous sections), and where appropriate coordinating with other child care services. As can be seen in the original tool, for each of the areas the professional must specify if the vulnerable situation is considered: i) non-existent (there are no indicators of risk of the vulnerable situation), ii) confirmed (there is clear evidence of it) or iii) suspect (if there is no conclusive evidence, but there are clear signs of risk that arouse suspicion). In addition, the professional must assess the severity of each of the vulnerable situations, taking into account the specific criteria detailed in the instrument, which are classified as: i) mild, ii) moderate, iii) severe or iv) very severe. These severity criteria were established by the professionals according to the degree of inappropriateness of the behaviour of the parents or legal guardians and the impact that the situation may have on the child at the physical, psychological, cognitive or social level.

Below, by way of example, is the description of neglect in terms of physical needs (food) together with the different severity classifications (see Table S2 for an example). The original instrument in Spanish contains a corresponding table for each of the areas of CM.

Since the authors who devised the tool indicate that some factors, such as the age of exposure, the frequency of exposure the support received, or the relationship with the aggressor, are crucial when it comes to understanding the impact that these situations have on the child, the protocol for the study included these variables in the summary table to be completed by the professionals.

Full instrument in Spanish available free at the following link:

<https://www.carm.es/web/pagina?IDCONTENIDO=9415&IDTIPO=246&RASTRO=c886$m5855>

**Table S2:** Example to assess physical needs (food) of physical neglect by TASSCV criteria.

| **PHYSICAL NEGLECT  IN TERMS OF PHYSICAL NEEDS – Food** | |
| --- | --- |
| 1. The child is seriously malnourished or dehydrated (serious weight loss, anaemia, etc.) and requires hospitalisation as a result. 2. The child is suffering from food poisoning (fever, vomiting, etc.) due to being given or allowed to eat food that is not suitable for a child of this age, that was not fit for human consumption or which had gone off. Hospitalisation is required. The provision or availability of this type of food is typical or frequent within the family setting. 3. The excessive amounts and unsuitable nature of the food given to the child have decisively led to obesity, which has caused serious health problems requiring hospitalisation. 4. A baby who is not fed regularly, is frequently given watered-down milk or is given some other type of nutritionally deficient food, showing symptoms of malnutrition or dehydration, even though only a recovery diet is required and hospitalisation is not necessary. | **Very severe** |
| 1. The child is suffering from some clinical symptoms of malnourishment, dehydration, food poisoning or being excessively overweight due to an unsuitable diet. The child needs medical attention or a rehabilitation diet; hospitalisation is not necessary. This represents, according to medical assessment, a serious risk for the child’s health if it is not corrected, though it is not life threatening. 2. A baby who is breast fed or receives some other type of nourishment and, according to medical assessment, requires milk or nutritional supplements that the baby is not receiving. The baby does not present symptoms of malnutrition or dehydration. | **Severe** |
| **1.** The child receives an insufficient amount of food or a diet which is nutritiously deficient. This has not led to any illness or important health effects. It is not the norm, but the child often does not receive at least one sufficiently nutritious meal a day. There is a clear need to correct the child’s diet, and there may be a medical recommendation to this effect. | **Moderate** |
| 1. Irregular meals that often are not even cooked, but the child normally receives one sufficiently nutritious meal a day. Sometimes a meal is missed. 2. Adequate diet but somewhat unbalanced in terms of nutrition. 3. The child is often left to get his or her own food, but sometimes only nutritiously inadequate food is available or there is not sufficient quantity of food available.   Although one or more of these circumstances holds, there has been no significant negative effect on the child’s health (according to medical opinion). A change in the diet or eating habits is advisable. | **Mild risk** |

**1.1. *Ad-hoc* hetero-administered questionnaire of maltreatment for children under 12**

Since the questionnaires used to assess childhood maltreatment (CM) are only validated for subjects older than 12 years old, we developed an *ad-hoc* questionnaire to be hetero-administered to the younger children (7 to 11). The participants answered “yes” or “no” to each of the questions and were asked to provide more detailed information where appropriate. The questionnaire consisted of 21 items classified into 5 types of CM (see Table S1).

**Table S1:** Items on the *ad-hoc* hetero-administered questionnaire to assess the maltreatment of children under 12

| Type of childhood maltreatment | Items |
| --- | --- |
| Physical neglect | Do you spend a lot of time alone, without being looked after by your parents/guardians or some other adult? |
|  | Have your parents/guardians ever not taken care of you when you were sick or not taken you to the doctor? |
|  | Do you think that you used not to dress as well as your classmates or be as clean and tidy as them because it was not important to your parents/guardians? |
|  | When you were little, did you have to get food for yourself because your parents/guardians were not at home or did not prepare food for you? Did that happen often to you? |
| Emotional neglect | Are your parents/guardians interested in what you get up to or who your friends are and how you are doing at school? |
|  | Do you feel that your parents/guardians ignore you and never give you hugs or kisses, or show you any other signs of affection? |
|  | Do you sometimes think that your parents/guardians don't know how to take care of you? |
|  | Have your parents/guardians ever showed no interest in you even when they knew that you needed help or support? |
| Emotional abuse | At home, have your parents/guardians ever told you that you are a nuisance and that they would be better off without you? |
|  | Have your parents/guardians ever said mean things to you, like that you are useless or ugly? |
|  | Do your parents/guardians ever call you names, tell you that you are worthless or make you feel really bad about yourself? |
|  | Do you think your parents/guardians ever treat you badly? |
| Physical abuse | Have your parents/guardians or some other adult ever shaken you, pinched, or burned you with a cigarette? |
|  | Have your parents/guardians ever shoved you, punched you up or kicked you hard? |
|  | Have you ever been hit with a belt, a broom, a stick or some other hard object? |
|  | Have you ever been threatened with a knife or other weapon? |
| Sexual  abuse | Has any family member or any adult shown you their private parts or touched them in front of you? |
|  | Have you ever been forced to take off your clothes or has anybody ever stared at your private parts? |
|  | Have you ever been made to touch or kiss someone’s private parts? |
|  | Has anyone ever tried to or actually touched your private parts? |
|  | Have you ever had full contact with anyone’s private parts? |

**1.3. Trier Social Stress Test for children (TSST-C)**

The Trier Social Stress Test for children (TSST-C) is the acute psychosocial stress protocol most widely used in children and adolescents. In what follows, we offer a detailed explanation of all its stages as applied in the present study. Upon arrival at the research center, each participant rested for 30 minutes in a quiet room accompanied by a familiar researcher (known from previous interviews). After this resting period, the participant entered a second room (the experimental room) where a panel of two unfamiliar judges wearing lab coats awaited sitting behind a table. The panel consisted of a woman and a man, so that the stress provoked by the TSST-C would not be affected by the gender of the judges. The judges then explained the nature of the tasks to the participant, highlighting that they would be videotaped so that experts would be able to analyze and rate their performance afterwards. At this point, the participants were also told that their performance would be compared with that of their peers and that they were expected to be the best. The judges were instructed to maintain a neutral stance throughout the TSST-C and to avoid giving any kind of positive feedback to the participants. During the first task (speech task), the participants were told the following story: “We were on holiday, in a village close to the sea. In the afternoon, we went to the beach to play with our friends. We played hide and seek, hit a ball around with bats, played in the sea and so on. One day I decided to swim to a small island that we could see from the beach. So, I swam and swam, and finally I arrived. I climbed up the cliff from the beach and when I reached the top, I discovered some huge leaves on the ground. When I lifted them up, underneath I saw…”. Then, they had 5 minutes to think of an ending to the story while the experts remained silent. Once the five minutes were over, one of the judges prompted the participant to stand in front of a microphone to finish the story, speaking freely for 5 minutes. Whenever a participant did not continue telling the story during this period, one of the judges let them know that they still had time to speak. Finally, the other judge introduced the second task (arithmetic task), consisting of a five-minute long serial subtraction. The task difficulty was adjusted according to age (subtracting 2 from 421 in children from 7 to 12 years old, and subtracting 3 from 758 in adolescents from 13 to 17 years old). Whenever a participant made a mistake, the judge asked them to start over. Including the instruction and both tasks, participants spent around 20 minutes in the experimental room. After the stress tasks were completed, the participant returned to the quiet room with the familiar researcher for an additional 30 minutes. After collection of all the TSST-C-related biological samples, the participants were debriefed about the true purpose of the TSST-C and the reasons why the judges were unfriendly. They were praised for their participation and told that their performance would never be examined or evaluated in the future.

All the participants were given a series of instructions to avoid factors that have been reported to influence cortisol levels. Specifically, they were told to refrain from eating or drinking (with the exception of water) for two hours before the TSST-C; to refrain from intense physical activity for 24 hours beforehand, and not to take benzodiazepine that day; if appropriate, to refrain from smoking for 1 hour before; not to consume alcohol or caffeine in the 24 hours preceding the TSST-C. Moreover, different variables previously reported to influence salivary cortisol levels after acute stress were recorded: time since the last meal, smoking, coffee and alcohol consumption, allergies, as well as the presence of acute infections.(Brigitte M. Kudielka, Hellhammer, & Wüst, 2009) The day of the menstrual cycle was also recorded for the female participants who had experienced menarche. Since the cortisol circadian rhythm is characterized by a peak in the morning and a progressive decline throughout the day, the TSST-C induces more reliable cortisol responses when conducted in the afternoon(B. M. Kudielka, Buske-Kirschbaum, Hellhammer, & Kirschbaum, 2004); hence, all the tests were scheduled at 4 pm.

**1.4. Salivary cortisol determination**

Cotton swabs for cortisol assessment were stored at -20ºC for a maximum of 3 months. The day of the cortisol determination, the tubes were thawed and centrifuged, following the manufacturer’s instructions, to remove debris from the saliva. After centrifuging and analyzing the samples, remaining saliva for future determinations was stored at -20ºC in an especial tube (cryovial) for a better preservation (avoiding biological degradation). Salivary cortisol concentration was determined using a high sensitivity enzyme-linked immunosorbent assay (ELISA) for cortisol (Salimetrics, LLC, State College, PA). Samples were tested in duplicate and the mean was calculated (μg/dL). Any cortisol concentrations with a coefficient of variation (CV%) higher than 30% were determined again. After reprocessing, only 1.9% of the total cortisol salivary samples had CV>30% (see Table S3 for details). All this samples were characterized by very low concentration (values ≤ 0.09μg/dL), being the majority samples in B4 (before bedtime) when cortisol levels are usually very low. Thus, all this samples were included in the final analysis after re-analyzing cortisol. Since all saliva samples were analyzed within the first three months after collection and the recruitment process spanned four years, multiple batches were used for the whole sample.

**Table S3**: Percentage of salivary cortisol samples with a coefficient of variation (CV%) higher than 30% included in the final analysis.

|  | Salivary cortisol samples with CV>30% | | |
| --- | --- | --- | --- |
|  | **n** | **% of the total salivary cortisol samples processed (n=1632)** | **% of the total salivary cortisol samples with CV>30% (n=31)** |
| Total diurnal salivary samples  (n=712) | 23 | 1.40% | 74.20% |
| B1 (n=178) | 1 | 0.06% | 3.22% |
| B2 (n=178) | 2 | 0.12% | 6.45% |
| B3 (n=178) | 3 | 0.18% | 9.68% |
| B4 (n=178) | 17 | 1.04% | 54.85% |
| Total TSST-C salivary samples  (n=920) | 8 | 0.49% | 25.80% |
| T1 (n=184) | 3 | 0.18% | 9.68% |
| T2 (n=184) | 0 | 0% | 0% |
| T3 (n=184) | 1 | 0.06% | 3.22% |
| T4 (n=184) | 0 | 0% | 0% |
| T5 (n=184) | 4 | 0.25% | 12.90% |
| TOTAL salivary cortisol samples  (n= 1632) | 31 | 1.89% | 100% |

1. **Supplementary results**
   1. **Sensitivity analysis including only subjects with confirmed history of CM**

To assess the possible biological differences between subjects with a confirmed and suspected history of CM, sensitivity analysis was performed considering only participants with clear evidence of a CM history from social services reports or family. Meanwhile, participants with only a suspected history of CM were considered as without CM. Comparing these results with those in the main report shows that confirmed and suspected participants with CM were characterized by similar HPA-axis disturbances.

**2.1.1. Diurnal salivary cortisol**

No interaction between CM and time was detected *(F*= 1.511, *p* = .212); however, the simple effects analysis revealed a significant time point-specific interaction between CM groups at B4 (before bedtime) (*F*= 3.897, *p* = .050). Although cortisol levels consistently decreased from lunchtime to bedtime in both groups, subjects with a confirmed history of CM were characterized by a more blunted decline, leading to a higher total hormonal output during the day, as evidenced by a higher AUCg, *F*(1,160) = 11.843, *p* = .001, *η_p_^2^* = .077. No significant differences were found with regard to AUCi, *F*(1,160) = 2.679, *p* = .104, *η_p_^2^* = .019. Hence, these findings highlight that participants with either confirmed or suspected CM were characterized by similar HPA-axis disturbances.

**2.1.2 Salivary cortisol response during the TSST-C**

A significant interaction was found between confirmed CM and time, *F*= 2.572, *p* = .039. Specifically, the simple effects analysis revealed a significant timepoint-specific interaction when comparing cortisol levels at T3 (immediately after the stressful situation), *F* = 6.386; *p* = .012, and at T4 (15 min after the stressful situation finished), *F* = 8.726, *p* =.003; while cortisol levels increased in non-maltreated individuals after acute stress, they remained constant in confirmed CM participants. Along these lines, subjects with confirmed CM showed lower levels of AUCi than those without CM, *F* (1,163) = 4.371, *p* = .038, *η_p_^2^* = .029, reflecting less variation in cortisol levels over time. However, confirmed CM was not associated with overall cortisol levels during the TSST-C, *F* = 3.754, *p* = .4054, or with AUCg levels, *F* (1,165) = .080, *p* = .778, *η_p_^2^* = .001. These findings highlight that confirmed and suspected cases of CM were characterized by similar HPA-axis alterations.

**2.1.3 Anxiety levels during the TSST-C**

The TSST-C consistently increased perceived anxiety in all the subjects, *F*= 32.551, *p* < .001. Furthermore, those with CM showed higher overall perceived anxiety during the entire procedure than subjects without CM, *F*= 20.644, *p* < .001. However, there were no interactions between time and CM (*F*= 0.411, *p* = .745), reflecting similar trajectories of perceived anxiety during the acute psychosocial stress in both subjects with and those without CM

**2.2. The effect of a current psychopathology, pubertal stage and sex on cortisol levels in confirmed and suspected CM subjects**

**2.2.1. Diurnal salivary cortisol**

Adolescents exhibited higher overall diurnal cortisol levels (AUCg) than children, *F* (1,160) = 7.697, *p* = .006, *η_p_^2^* = 0.051. Furthermore, there was a significant interaction between pubertal stage and current psychopathological status with regard to AUCg, *F* (1,160) = 5.594, *p* = .019, *η_p_^2^* = 0.038, and AUCi (*F* (1,160) = 7.844, *p* = .006, *η_p_^2^* = 0.052. Indicating that children, regardless if they have a current psychiatric disorder, showed similar cortisol fluctuation during the day (AUCi) and similar total cortisol output over a day (AUCg). However, while healthy adolescents exhibited higher levels of cortisol at the morning decreasing much more their cortisol levels from morning to lunchtime, adolescents with current psychopathology showed less cortisol levels at the morning and higher cortisol levels at night, decreasing less their cortisol levels from midday to bedtime (See Figure S1).

**
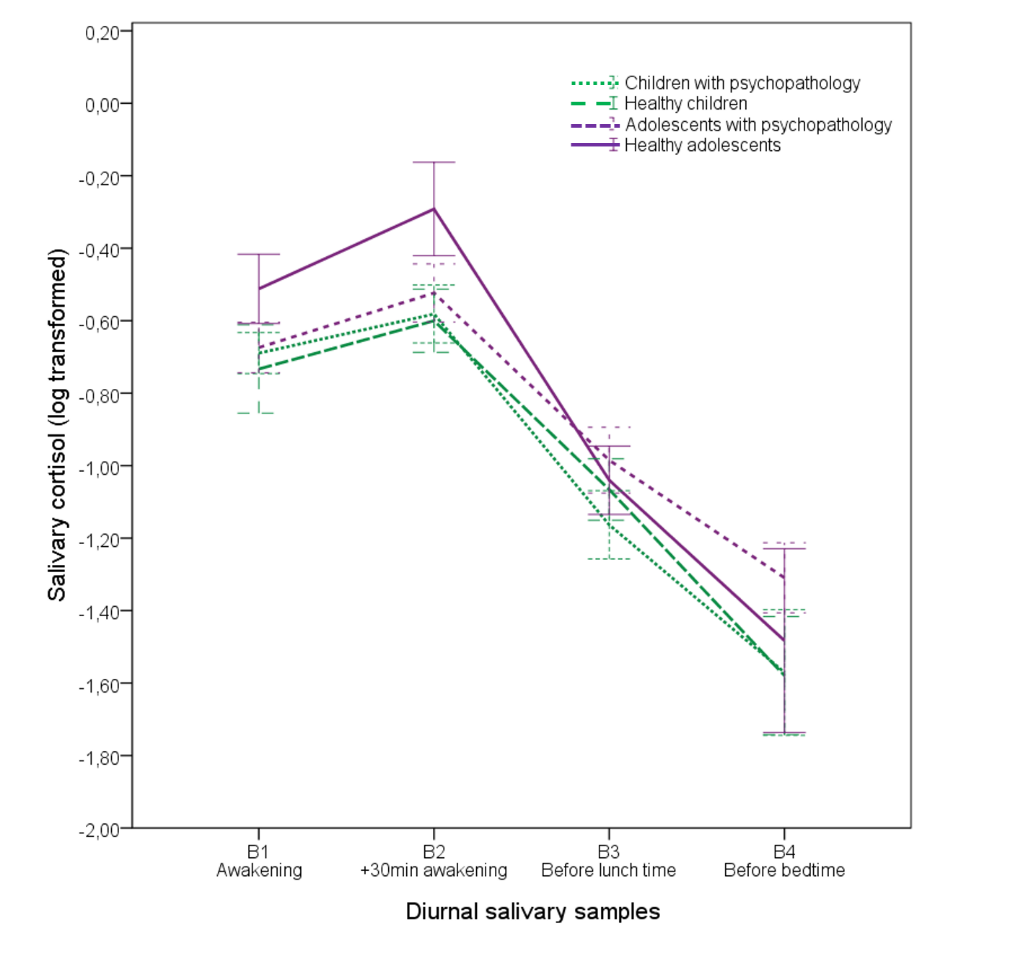
Figure S1:** Diurnal cortisol levels in children and adolescents with and without current psychiatric disorder.

Moreover, there was a significant interaction between pubertal stage and sex in AUCi levels, *F* (1,160) = 4.906, *p* = .028, *η_p2_* = 0.033. Such that all male, both children and adolescents, showed similar diurnal cortisol fluctuation, greater differences were observed when comparing children and adolescents in the female group. While female adolescents exhibited the highest AUCi values, female children showed the lowest AUCi levels. Specifically, it seems that female adolescents showed higher cortisol levels at the morning, showing a more pronounced decrease in cortisol levels from morning to lunchtime.

**2.2.2. Cortisol reactivity during the TSST-C**

Subjects with a current psychiatric diagnostic had lower overall cortisol levels (AUCg) during the TSST-C than those without a psychopathology, *F* (1,165) = 6.151, *p* = .014, *η_p_^2^* = .040. Moreover, adolescents had higher overall levels of cortisol (AUCg) during the TSST-C than children, *F*(1,165) = 25.649, *p <* .001, *η_p_^2^* = .148. However, no significant differences were found in any of these variables (sex, pubertal stage and current psychiatric disorder) in the cortisol levels fluctuation during the TSST-C.


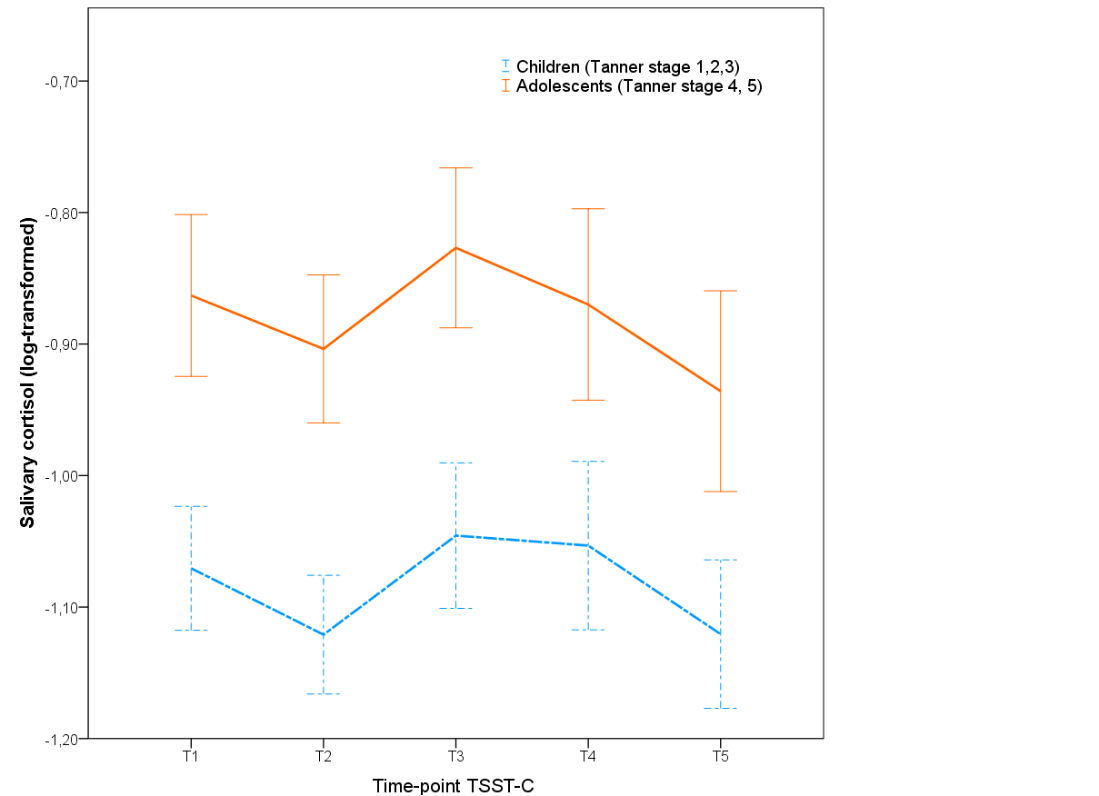
**Figure S2:** Cortisol levels during TSST-C in children and adolescents

**2.2.3 Anxiety levels during the TSST-C**

Girls showed increased levels of anxiety perception throughout the whole TSST-C, compared with boys, *F* (1,160) = 10.852, *p* = .001, *η_p_^2^* = .071. Nevertheless, no significant differences were found in in the magnitude of the increase of the anxiety perception during the TSST-C in any of these variables (sex, pubertal stage and current psychiatric disorder).

**2.3. Sensitivity analysis classifying the groups as low/high CM depending on severity/frequency**

The effects of CM were re-analyzed according to its severity and frequency, following a new categorization of these variables. Specifically, both severity and frequency were dichotomized into low and high exposure groups: participants without CM were grouped together either with subjects exposed to mild experiences of CM (low severity group) or with subjects who had been exposed to CM just once or sometimes (low frequency group). The high exposure group was defined by either moderate-to-severe exposure or often-frequent exposure.

**2.3.1. Diurnal salivary cortisol**

No significant differences were observed in overall diurnal cortisol levels according to either severity, *F_severity_*= 1.182, *p*= . or frequency of the exposure to CM, *F_frequency_*= 0.633, *p* = 427. However, the high exposure groups was characterized by a significant increase in AUCg, *F_severity_*(1, 160) = 7.197, *p* = .008, *η_p_^2^* = .048; *F_frequency_*(1, 1603) = 5.612, *p* = .019, *η_p_^2^* = .038, indicating higher cortisol levels during the day. Moreover, no global interaction was detected between time and the high/low maltreatment severity or frequency groups, *F_severity_*= 1.921, *p* = .127; *F_frequency_* = 1.519, *p* = .211or in AUCi levels, *F_severity_* (1, 160) = 2.467, *p* = .118, η_p2_ = .017; *F_frequenc_*_y_(1, 160) = 1.186, p = .278, ηp2 = .008, reflecting a similar diurnal trajectory in both groups. However, the simple effects analysis revealed a significant time point-specific interaction between CM groups at B4 (before bedtime) (*F*_severity_= 4.676, *p*_severity_ = .032).; although cortisol levels consistently decreased from morning to lunchtime in both groups, the high frequency group was characterized by a more blunted decline.

**2.3.2. Cortisol reactivity during the TSST-C**

Following the dichotomization of low/high groups of severity or frequency, both were associated with time-specific cortisol values, *F_severity_* = 4.613, *p* = .001; *F_frequency_* = 4.367, *p* = .002. Specifically, the simple effects analysis revealed a significant timepoint-specific interaction when comparing cortisol levels at T3 (immediately after the stressful situation), *p_severity_* = .008; *p_frequency_*= .017, at T4 (15 min after the stressful situation finished, *p_severity_* = .001; *p_frequency_*= .002, and at T5 (30 min after the stressful situation finished), *p_severity_* = .048; *p_frequency_*= .039. Low-exposure participants were characterized by an increase in cortisol values after acute stress, while high-exposure participants exhibited a decrease in cortisol. In line with this, participants in the high severity or high frequency group showed lower values of AUCi, *F_severity_* (1,165) = 8.306, *p* = .005, *η_p_^2^* = .053; *F_frequency_* (1,165) = 6.356, *p* = .013, *η_p_^2^* = .041. No significant differences were found in overall cortisol levels during the TSST-C, evidencied by AUCg, *F_severity_* (1,165) = 0.002, *p* = .963, *η_p_^2^* < .001; *F_frequency_* (1, 165) = 0.243, *p* = .623, *η_p_^2^* = .002.

**2.4.** **Relationship between anxiety perception and cortisol secretion**

**2.4.1 Diurnal salivary cortisol**

Non-significant correlation between anxiety trait and overall diurnal cortisol levels (AUCg) was found in subjects with CM (r= .227, p = .227), neither in participants without history of CM (r= .089, p =.448).

**2.4.2 Cortisol reactivity during the TSST-C**

Subjects with CM did not show a significant correlation between their stress perception and their cortisol levels at any of the TSST-C timepoints (see Figure S1a). In contrast, those without CM exhibited a significant negative correlation between their stress perception and their cortisol levels at different timepoints during the TSST-C (see Figure S1b). Specifically, stress perception at T1 was negatively associated with cortisol levels at T3 (r = -.387, p < .001), T4 (r = -.300, p = .004) and T5 (r = -.355, p = .001). Similarly, the stress perception at T2 was negatively associated with cortisol levels at T3 (r = -.425, p < .001), T4 (r = -.360, p = .001) and T5 (r = -.343, p = .001). Finally, the stress perception at T5 was associated with cortisol levels at T5 (r = -.457, p < .001). These findings indicate that in subjects without CM a lower anxiety perception prior to stress exposure was associated with a greater increase in cortisol after acute stress, while higher perceptions of anxiety were associated with lower levels of cortisol after the stress task.

**Figure S3:** Relationship between anxiety perception and cortisol secretion during the TSST-C in maltreated and non-maltreated subjects. Maltreated subjects showed no correlation between their stress perception and their cortisol levels at any of the timepoints. However, non-maltreated subjects exhibited negative correlation between their anxiety perception and cortisol levels at different timepoints during the TSST-C.


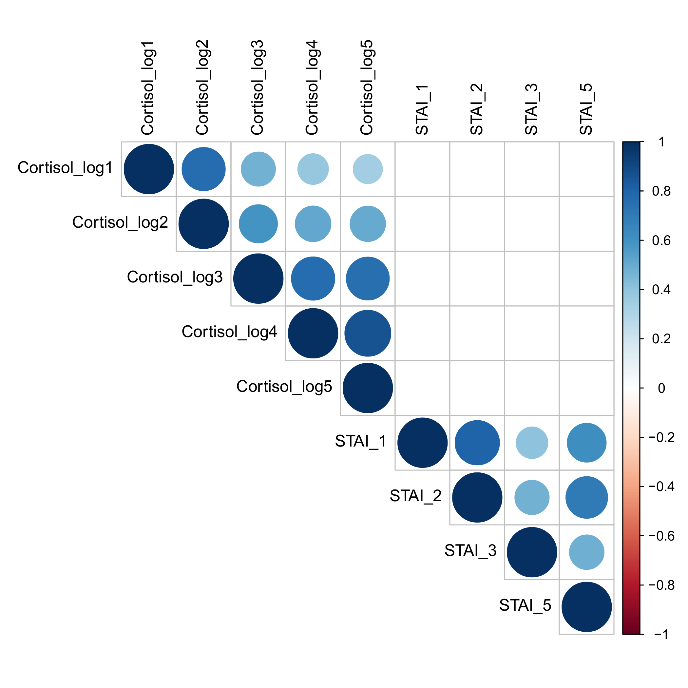

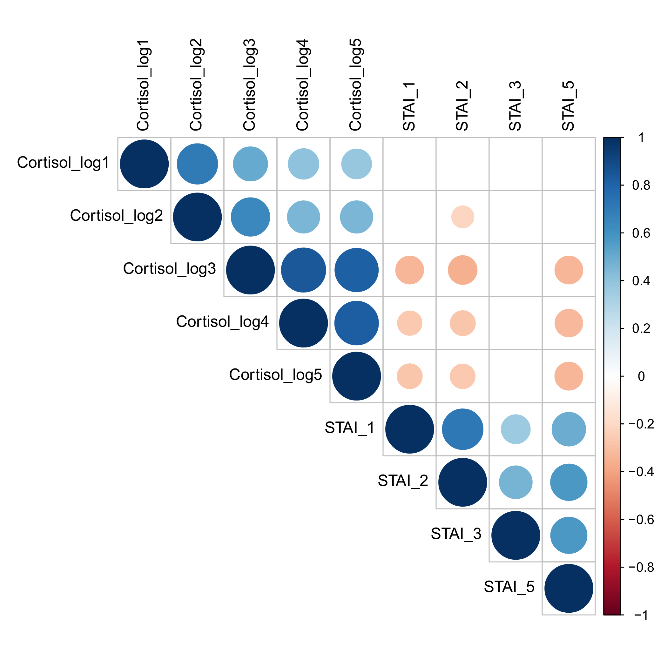
**Youth without a history of maltreatment Youth with a history of maltreatment**

1. **Supplementary histograms**

**Figure S4:** Histograms of age and pubertal state (Tanner stage)


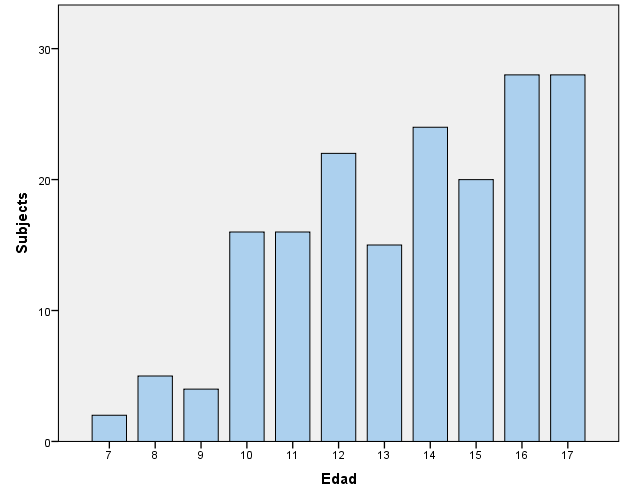


**Age**


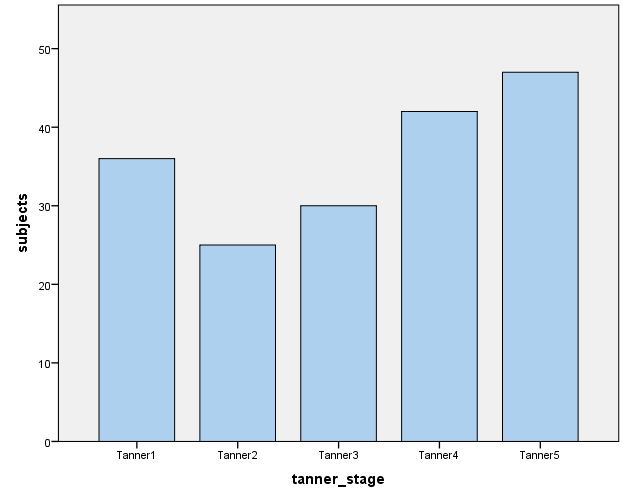


**Tanner stage**

**Figure S5:** Histograms of cortisol concentration of diurnal salivary samples (μg/dL, log-transformed values)


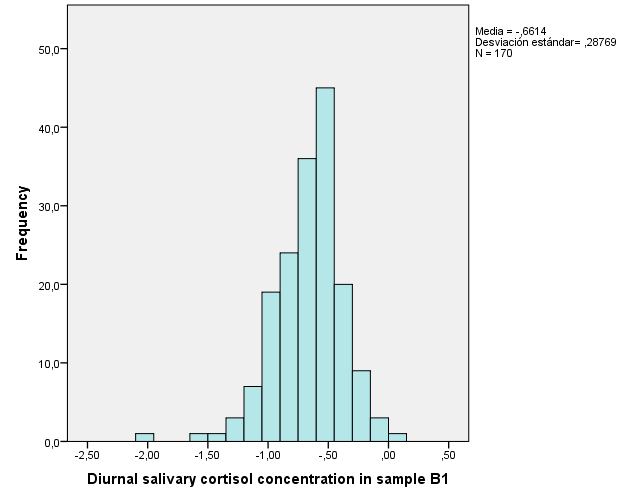

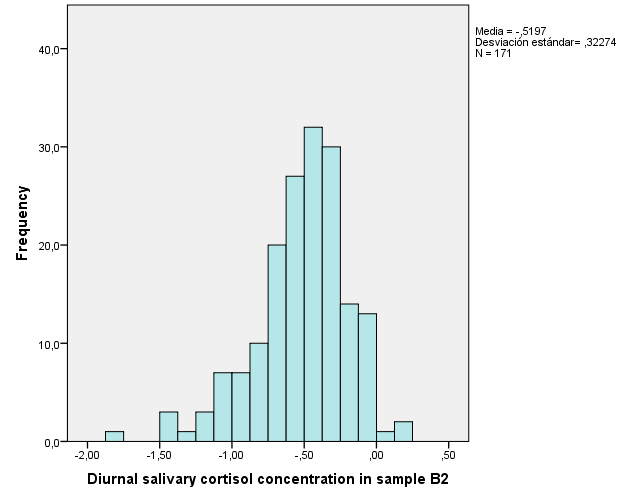


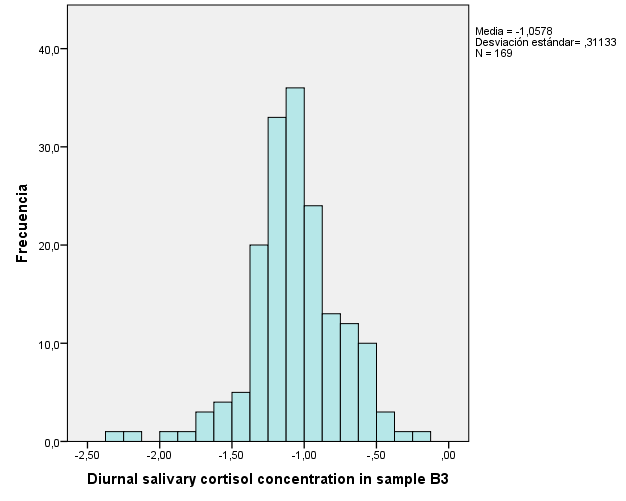

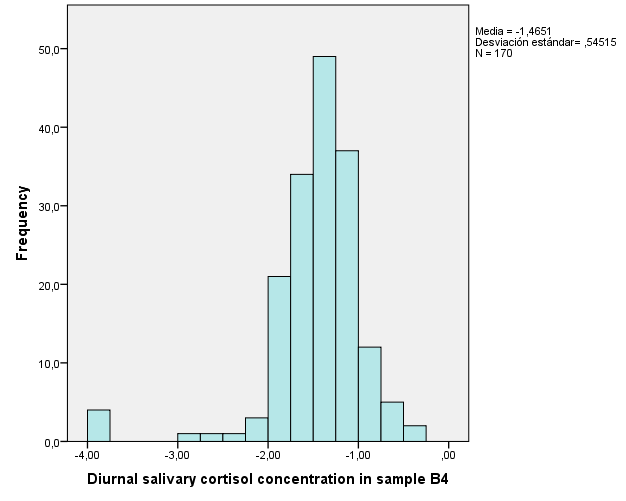


**Figure S6:** Histograms of cortisol concentration of salivary samples during TSST-C (μg/dL, log-transformed values)


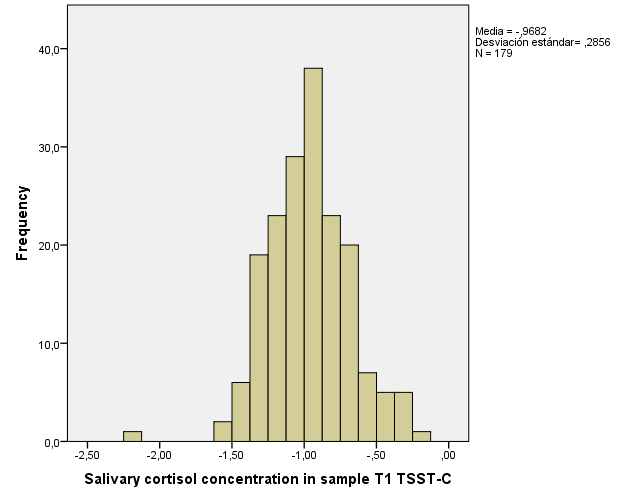

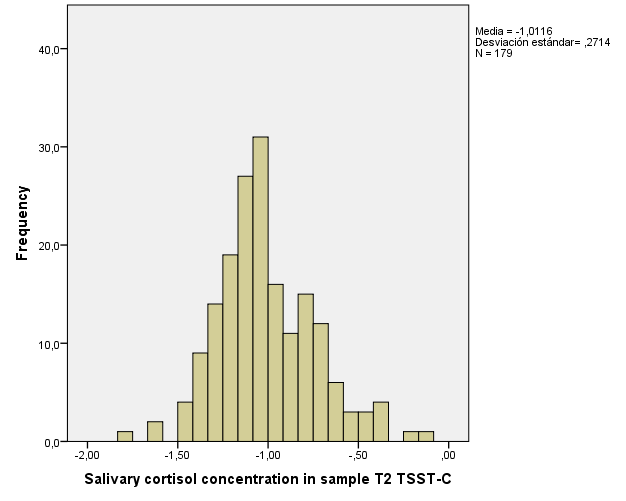


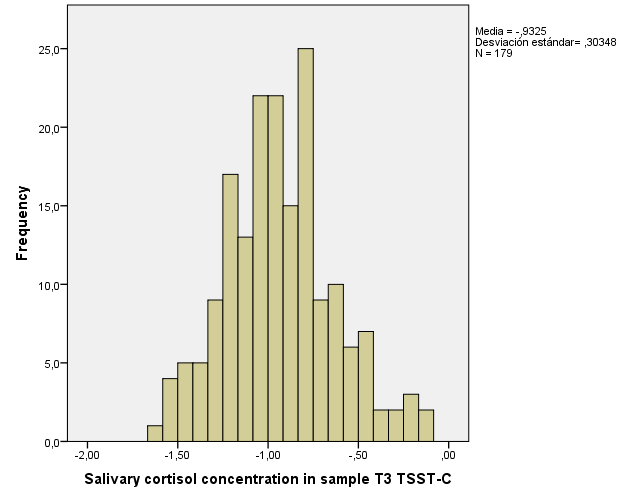

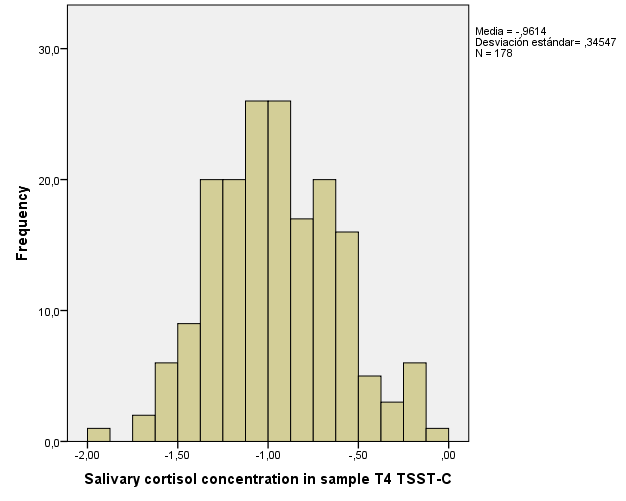


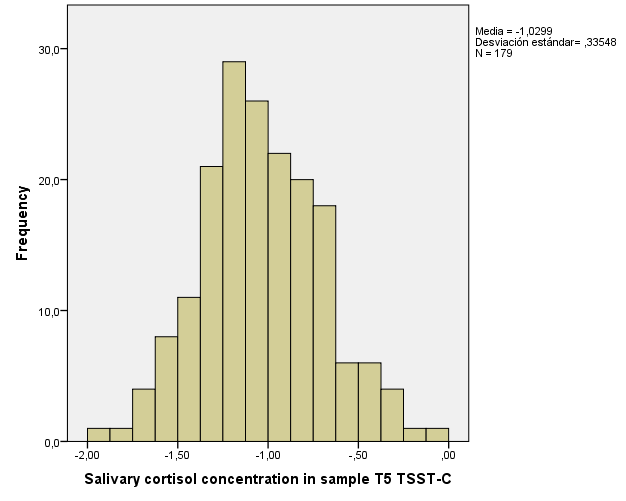


1. **Supplementary references**

CARM. (2012). Instrumento para la valoración de la gravedad de las situaciones de desprotección infantil (Tool for assessing the severity of situations in which children are vulnerable- TASSCV). *Servicios Sociales de Atención Primaria y Especializados de La Región de Muricia*.
https://www.carm.es/web/pagina?IDCONTENIDO=9415&IDTIPO=246&RASTRO=c886$m5855

Kudielka, B. M., Buske-Kirschbaum, A., Hellhammer, D. H., & Kirschbaum, C. (2004). HPA axis responses to laboratory psychosocial stress in healthy elderly adults, younger adults, and children: Impact of age and gender. *Psychoneuroendocrinology*, *29*(1), 83–98. https://doi.org/10.1016/S0306-4530(02)00146-4

Kudielka, Brigitte M., Hellhammer, D. H., & Wüst, S. (2009). Why do we respond so differently? Reviewing determinants of human salivary cortisol responses to challenge. *Psychoneuroendocrinology*, *34*(1), 2–18. https://doi.org/10.1016/j.psyneuen.2008.10.004
